# Wheat fiber mitigates colitis via non-SCFA microbial metabolite-trained intestinal macrophages

**DOI:** 10.1126/sciadv.aec5757

**Published:** 2026-03-25

**Authors:** Seong-eun G. Kim, Rachael Ott, Alexis Bretin, Hirohito Abo, Yanling Wang, Yadong Wang, Shawn Winer, Daniel A. Winer, Lavanya Reddivari, Stacey L. Heaver, Ruth E. Ley, Michael Pellizzon, Vu L. Ngo, Andrew T. Gewirtz

**Affiliations:** ^1^ Center for Inflammation, Immunity and Infection, Institute for Biomedical Sciences, Georgia State University, Atlanta, GA 30303, USA.; ^2^ Department of Laboratory Medicine and Pathobiology, University of Toronto, Toronto, ON M5S 3K3, Canada.; ^3^ Buck Institute for Research on Aging, Novato, CA 94945, USA.; ^4^ Division of Cellular & Molecular Biology, Diabetes Research Group, Toronto General Hospital Research Institute (TGHRI), University Health Network, Toronto, ON M5G 1L7, Canada.; ^5^ Department of Immunology, University of Toronto, Toronto, ON M5S 1A8, Canada.; ^6^ Department of Food Science, Purdue University, West Lafayette, IN 47907, USA.; ^7^ Department of Microbiome Science, Max Planck Institute for Biology, Tubingen 70206, Germany.; ^8^ Research Diets Inc., New Brunswick, NJ 08901, USA.

## Abstract

The advent of highly refined wheat products has reduced fiber consumption, which is associated with increased risk for inflammatory bowel disease (IBD). We found that enriching diets with wheat fiber (WF) protected mice against colitis, especially relative to a low-fiber diet, as assessed by clinical, histopathologic, morphologic, and immunologic parameters. WF’s protection against colitis was independent of short-chain fatty acids (SCFAs) yet associated with preservation of microbiota diversity, including maintenance of *Bacteroides thetaiotaomicron* (*B. theta*), which was necessary and sufficient for WF’s colitis protection. *B. theta*’s presence in gnotobiotic mice resulted in WF-induced fecal metabolites that reprogrammed macrophages toward an M2-like phenotype. Metabolic and phenotypic reprogramming of macrophages ex vivo via WF-induced metabolites, followed by their transplantation into mice, recapitulated WF’s protection against colitis. Thus, microbiota-mediated metabolism of WF promotes macrophages that reduce proneness to intestinal inflammation, suggesting a mechanism by which WF consumption may curb development of IBD.

## Teaser

Wheat fiber mitigates colitis by preserving *B. thetaiotaomicron*, whose metabolites train macrophages to reduce gut inflammation.

## INTRODUCTION

Industrialization ushered changes in food production and dietary practices that resulted in reduced levels of dietary fiber consumption. Consequently, most people in developed countries do not meet recommendations by health agencies such as the US Department of Agriculture (USDA) and World Health Organization that individuals should consume a minimum of 25 to 38 g of fiber per day (*1*). Such recommendations are based largely on epidemiological evidence that consumption of plant-based foods naturally rich in fiber associates with good health and interventional studies that found that increasing consumption of such foods improved numerous health outcomes (*2*). The extent to which benefits of fiber-rich foods actually result from their fiber content per se, and, furthermore, underlying mechanisms, are largely derived from animal models, wherein fiber content can be specifically manipulated although doing so requires use of compositionally defined diets (CDDs) that are made from highly refined ingredients rather than standard grain-based chow (GBC), which is a conglomeration of fiber-rich plant products that vary seasonably and, moreover, cannot be manipulated (*3*). This caveat notwithstanding, animal studies not only support the general notion that dietary fiber promotes intestinal and metabolic health but also highlight that fibers are a very broad class of molecules with distinct structures and properties and, consequently, distinct impacts on the intestinal microbiota and host health (*4*). For example, enriching low-fiber (LF) diets with inulin, derived from chicory root, results in it being fermented by microbiota to short-chain fatty acids (SCFAs), increasing microbiota density, which results in enhanced enterocyte proliferation and increased interleukin-22 (IL-22) expression (*5*). Collectively, such events result in resistance to diet-induced obesity (*5*) but predispose mice to severe dextran sulfate sodium (DSS)–induced colitis (*6*). In contrast, psyllium fiber, derived from Plantago seeds, lowers bacterial density, IL-22 expression, and SCFA levels but nonetheless results in a microbiota-dependent increase in serum bile acids and, moreover, an farnesoid X receptor (FXR)–mediated protection against colitis (*7*).

The contrasting actions of inulin and psyllium, both of which are widely consumed by humans as supplements, led us to appreciate the need to examine impacts of the fibers that became less frequently consumed by humans as the prevalence of inflammatory bowel disease (IBD), and other chronic inflammatory diseases, increased. Breads, and other flour-based foods, which had long been nearly exclusively made from whole wheat kernels, are rich in fiber (*8*). Most of this fiber is absent in “white” breads, which are made from the wheat kernel’s endosperm, the isolation of which was made practical by industrialization (*9*). Hence, we sought to investigate the impact of wheat fiber (WF) on experimental colitis and, furthermore, discern the mechanism(s) that underlie its impacts. We found that enriching an LF diet with WF protected mice against colitis. Such protection was not mediated by SCFA, IL-22, or FXR activation. Rather, WF promoted microbiota diversity, including maintaining bacteria such as *Bacteroides thetaiotaomicron* (*B. theta*), which is rich in carbohydrate-active enzymes (CAZymes) (*10*), that metabolized WF to yield products that reprogrammed intestinal macrophages, thereby protecting against colitis.

## RESULTS

### WF protects mice against DSS colitis independent of SCFAs

**Fig. 1.**
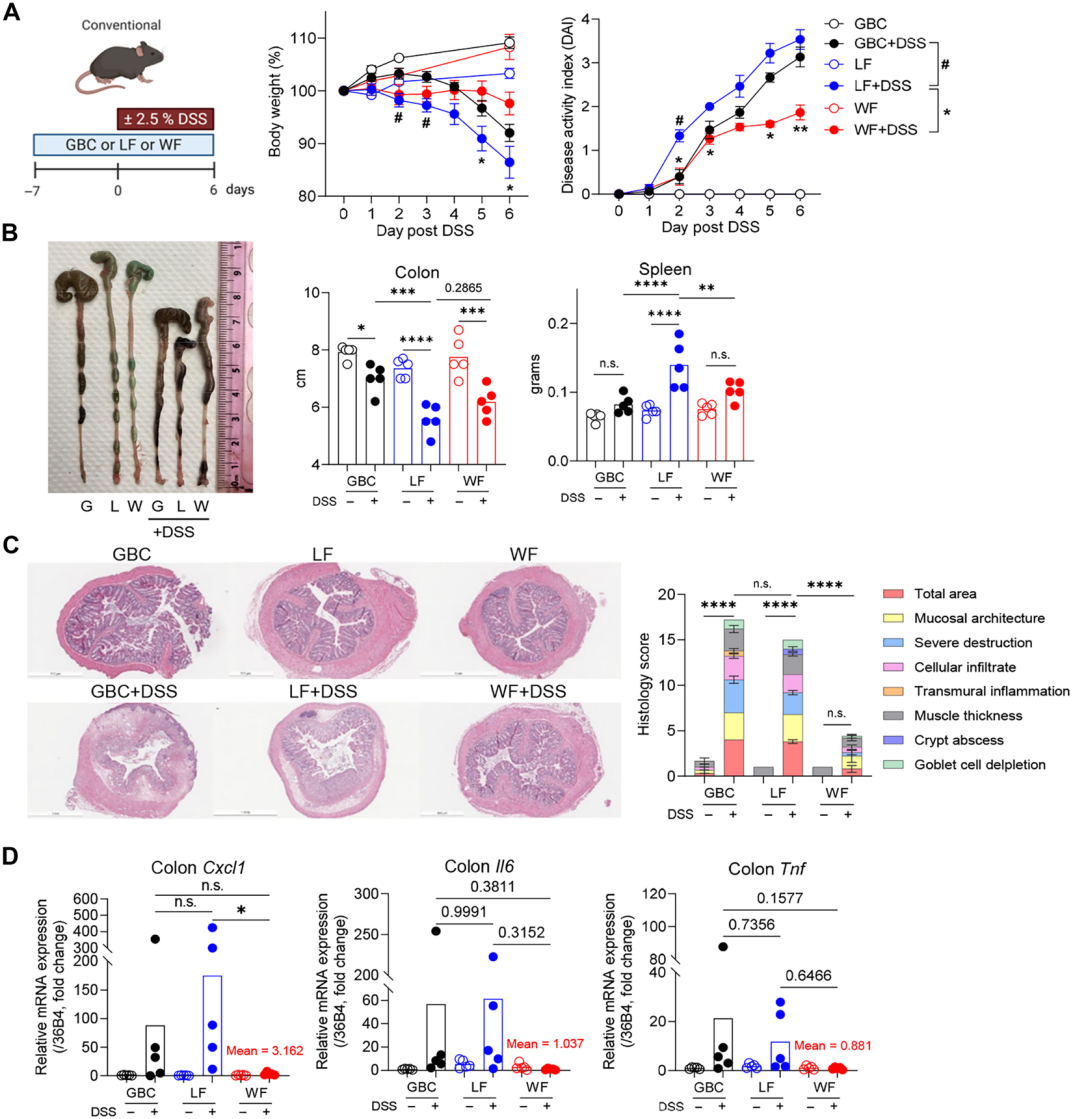
WF protected mice against DSS-induced colitis. Seven-week-old female B6 mice (*n* = 5 per group) were fed GBC, LF, or WF and given either water or DSS water for 6 days. (**A**) Experimental design, body weight loss, and DAI post–DSS administration (#: GBC+DSS versus LF+DSS; *: WF+DSS versus LF+DSS). (**B**) Representative colon image, colon length, and spleen weight following DSS treatment. (**C**) Representative colon histology images and blind scoring (*n* = 3 per non-DSS groups; *n* = 5 per DSS groups). (**D**) Colon cytokine levels measured by RT-qPCR. The results are representative of three independent experiments using male and female mice. All data are presented as mean values ± SEM. *n* = 5 per group unless otherwise indicated. Statistical evaluation was conducted by two-way ANOVA (*P* < 0.0001) followed by Dunnett’s multiple comparisons test (versus LF+DSS) for (A) and one-way ANOVA followed by Sidak’s multiple comparisons test for (B), (C), and (D). n.s. (not significant), *P* > 0.05; *P < 0.05; **P < 0.01; ***P < 0.001; ****P < 0.0001. Schematics were created with BioRender.com. Gewirtz, A. (2026) https://BioRender.com/ze78l8j.

Six-week-old C57BL/6 mice, which had been maintained on GBC, remained on this standard rodent diet or were switched to an open-source CDD that commonly serves as both a control diet and/or an LF diet (5% cellulose, w/w) in nutrition studies or a CDD enriched with cellulose, oat fiber, or WF such that the total fiber content was ∼15% by weight (Fig. 1 and fig. S1A) and thus similar to GBC, the fiber content of which varies from about 15 to 25% across batches (*11*). The precise composition of each CDD used is described in Table 1. One week following diet switch, mice were administered drinking water containing 2.5% DSS water to induce colitis. Colitis was monitored by clinical-type parameters (body weight, stool consistency, and fecal blood) until one or more mice displayed 20% weight loss, at which time mice were euthanized and colitis severity further assayed morphologic, immunologic, and histopathologic readouts. In accord with previous studies (*6*, *7*), mice fed the LF diet developed more severe disease than GBC-fed mice (Fig. 1, A to D, and fig. S1A). Colitis severity was only modestly affected by enrichment of the LF with cellulose or oat fiber (fig. S1A). In contrast, all parameters assayed indicated that mice fed the WF diet exhibited only mild disease prompting a more in-depth study of this fiber.

**Table 1.** Diet compositions.

	LF		Cellulose		WF		Oat^min^		Oat^high^	
Research Diets catalog no.	D12450J		D13081109		D20030503		D20030501		D20030502	
	50 g cellulose/4057 kcal		200 g cellulose/4057 kcal		50 g cellulose+150 g WF/4057 kcal		50 g cellulose+150 g Oat Fiber 401/4057 kcal		50 g cellulose+150 g Oat Fiber 600/4057 kcal	
	**g %**	**kcal %**	**g %**	**kcal %**	**g %**	**kcal %**	**g %**	**kcal %**	**g %**	**kcal %**
Protein	19	20	17	20	17	20	17	20	17	20
Carbohydrate	67	70	59	70	59	70	71	70	59	70
Corn starch	48	50	42	50	42	50	42	50	42	50
Fat	4	10	4	10	4	10	4	10	4	10
Total		100		100		100		100		100
										
**Ingredient**	**g**	**kcal**	**g**	**kcal**	**g**	**kcal**	**g**	**kcal**	**g**	**kcal**
Casein	200	800	200	800	200	800	200	800	200	800
l-Cystine	3	12	3	12	3	12	3	12	3	12
Corn starch	506.2	2025	506.2	2025	506.2	2025	506.2	2025	506.2	2025
Maltodextrin 10	125	500	125	500	125	500	125	500	125	500
Sucrose	68.8	275	68.8	275	68.8	275	68.8	275	68.8	275

Cellulose, BW200	50	0	200	0	50	0	50	0	50	0
Oat Fiber 401, Vitacel	0	0	0	0	0	0	150	0	0	0
Oat Fiber 600, Vitacel	0	0	0	0	0	0	0	0	150	0
WF 600, Vitacel	0	0	0	0	150	0	0	0	0	0

Lard	20	180	20	180	20	180	20	180	20	180
Soybean oil	25	225	25	225	25	225	25	225	25	225
Mineral Mix S10026	10	0	10	0	10	0	10	0	10	0
Dicalcium phosphate	13	0	13	0	13	0	13	0	13	0
Calcium carbonate	5.5	0	5.5	0	5.5	0	5.5	0	5.5	0
Potassium citrate, 1 H_2_O	16.5	0	16.5	0	16.5	0	16.5	0	16.5	0
Vitamin Mix V10001	10	40	10	40	10	40	10	40	10	40
Choline bitartrate	2	0	2	0	2	0	2	0	2	0
Yellow Dye #5, FD&C	0.04	0	0	0	0	0	0.05	0	0.025	0
Red Dye #40, FD&C	0	0	0	0	0.05	0	0	0	0.025	0
Blue Dye #1, FD&C	0.01	0	0.05	0	0	0	0.01	0	0	0
Total	1055.05	4057	1205.05	4057	1205.05	4057	1205.05	4057	1205.05	4057
kcal/g	3.85		3.37		3.37		3.37		3.37	
	**g**	**g %**	**g**	**g %**	**g**	**g %**	**g**	**g %**	**g**	**g %**
Total fiber	50	4.7	200	16.6	189.1	15.7	180.1	14.9	190.3	15.8
Insoluble fiber	50	4.7	200	16.6	185.2	15.4	178.4	14.8	181.4	15.1
Soluble fiber	0	0	0	0	3.9	0.3	1.7	0.1	8.9	0.7
Oat Fiber 401: 85.6% insoluble, 1.1% soluble, total 86.7% fiber; Oat Fiber 600: 87.6% insoluble, 5.9% soluble, total 93.5% fiber; WF: 90.1% insoluble, 2.6% soluble, total 92.7% fiber.										

Switching mice from GBC to the LF diet by itself (i.e., in the absence of DSS) results in reduced colonocyte proliferation, which overtly manifests as a stark reduction in colon weight and a mild colonic shortening, suggesting low-grade inflammation (*5*). Such impacts of the LF diet were partially alleviated by WF (Fig. 1B and fig. S1B) albeit to a lesser extent than what we previously observed for inulin or psyllium (*5*, *7*). Benefits of WF were much clearer following DSS treatment wherein mice consuming the WF diet exhibited milder colitis compared to not only those fed the LF CDD but also those fed GBC (Fig. 1, A to D). WF-fed mice exhibited a particularly stark reduction in DSS-induced pro-inflammatory cytokine expression (Fig. 1D), suggesting that WF may have immune modulatory properties that protect against intestinal inflammation.

To probe the mechanism by which WF protected against colitis, we first examined its physiochemical properties and chemical nature. Initial analysis indicated that the specific WF we used was 92.7% fiber (w/w), of which 97% was insoluble (Table 1). Carbohydrate analysis found that, in contrast to previously reported analyses of WF but aligning with its lack of solubility, our WF was low in arabinoxylan and predominantly comprised xylan (fig. S1C). WF’s lack of solubility suggested that it would not be easily fermentable (*12*) and thus would not elicit increases in SCFAs. Analysis of cecal SCFAs generally supported this notion in that, in contrast to inulin, the WF did not result in increases in butyrate although a modest increase in acetate was observed in WF-fed mice (fig. S1D), suggesting the possibility that some portion of WF might be fermented. In any event, WF’s protection against DSS colitis was not impeded by hops beta acids, which block SCFA production (*5*, *13*), arguing against the notion that fermentation of WF contributes to its prevention of colitis (fig. S1E). Another candidate mechanism we considered was IL-22, which is induced by inulin (*5*) and can protect mice from DSS-induced colitis (*14*), but quickly ruled out a role for IL-22 after observing that addition of WF did not increase IL-22 expression (fig. S1F). Last, we considered the possibility that the FXR bile acid receptor, which mediates psyllium’s anti-inflammatory action (*7*), might play a role but found that WF’s protection against DSS colitis was maintained in FXR-deficient mice arguing against this possibility (fig. S1G).

### WF preserves gut microbiota diversity, including *B. theta*, which mediates protection against colitis

**Fig. 2.**
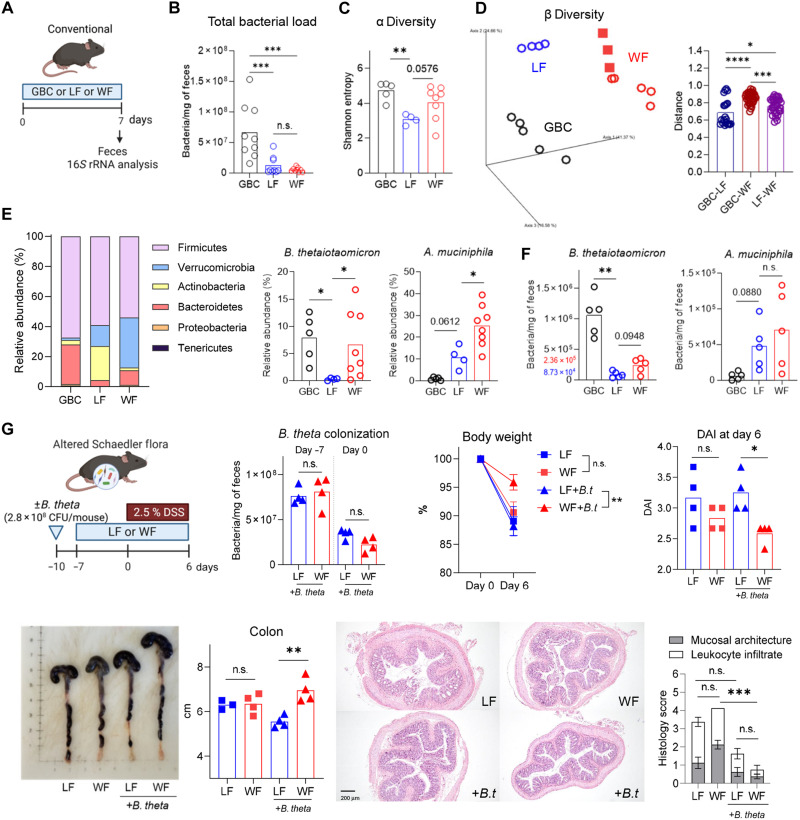
WF-induced gut microbiota diversity was necessary for colitis protection, and *B. thetaiotaomicron* was sufficient to mediate the protection. (**A**) Schematic for (B) to (F). B6 conventional mice were fed GBC, LF, or WF for a week (*n* = 5/GBC, *n* = 4/LF, and *n* = 8/WF), and the feces were analyzed by qPCR or 16*S* rRNA gene sequencing using iSeq. (**B**) Total bacterial load analyzed by qPCR using the 16*S* universal primer (*n* = 9 per group). (**C**) Alpha diversity. (**D**) Beta diversity (Bray-Curtis) and distance between the groups. Data from WF-fed mice in two separate cohorts (red circles and squares) were included to account for cage effects. (**E**) Taxonomy analysis at the phylum level and relative abundance of representative species. (**F**) In a separate cohort (*n* = 5 per group), the absolute abundance of *B. thetaiotaomicron* and *A. muciniphila* was confirmed by qPCR using bacteria-specific primers. (**G**) B6 ASF mice (*n* = 4 per group) were either gavaged with or without *B. theta* (*B.t*) and then fed LF or WF for a week, followed by DSS water for 6 days. Bacteria colonization, weight loss, and DAI were measured. Representative colon images for colon length, and representative colon histology image and scoring. The results are representative of three independent experiments using male and female mice. All data are presented as mean values ± SEM. Statistical evaluation was conducted by one-way ANOVA (B: *P* = 0.0001; D: *P* < 0.0001; G: DAI, *P* = 0.0278; colon length, *P* = 0.0038; histology, *P* = 0.0004) or two-way ANOVA (G: body weight, *P* = 0.0596) followed by Tukey’s multiple comparisons test or multiple *t* tests (two prespecified pairwise comparisons were assessed using unpaired two-tailed Welch’s *t* tests) followed by Bonferroni correction (C, E, and F). n.s. *P* > 0.05; *P < 0.05; **P < 0.01; ***P < 0.001; ****P < 0.0001. Schematics were created with BioRender.com. Gewirtz, A. (2026) https://BioRender.com/g9ryi1a.

We next examined whether WF’s prevention of colitis was mediated by microbiota. In accord with previous studies, switching mice from GBC to an LF diet resulted in stark changes in the fecal microbiome, including reductions in bacterial density and alpha-diversity as well as an overall change in taxonomic composition (Fig. 2A). WF did not affect bacterial density but nonetheless had marked changes in its diversity and composition (Fig. 2, B to E). The change in the microbiome that resulted from WF enrichment was greater than the change that resulted from switching from GBC to the LF diet. Principal coordinates analysis (PCoA), particularly that the distance of GBC-WF was greater than GBC-LF, argued against the notion that WF had broadly restored the gut microbiome toward that of GBC-fed mice (Fig. 2D). Rather, WF enrichment of diet resulted in preservation of select taxa such as *B. theta* and further increases in select taxa such as *Akkermansia muciniphila* (*A. muc*), which had been enriched by the LF diet (Fig. 2E and fig. S2). Measuring these taxa by quantitative polymerase chain reaction (qPCR) in a distinct cohort of mice found a similar pattern of change (Fig. 2F). Both *B. theta* and *A. muc* are known to use complex carbohydrates and reported to alleviate DSS colitis in GBC-fed mice (*15*–*17*), suggesting that their enrichment by WF may have contributed to the protection it conferred.

To gain insights into the functional consequences of WF’s impact on the microbiota, we turned to predicted metagenome analysis using PICRUSt2 (figs. S3 and S4). Enriching the LF diet with WF shifted the overall enzyme commission (EC) profile toward that of the GBC group (fig. S3A). More specifically, WF supplementation increased the predicted abundance of glycoside hydrolases (GHs) targeting plant polysaccharides (fig. S3B), including xylan and cellulose, which were abundant in WF (fig. S1C). Relative contribution analysis highlighted relevant predicted function-taxa association, such as xylan-degrading *Lachnospiraceae* (*18*, *19*) (fig. S4A, pink) or starch-degrading *Bacteroides* (*20*, *21*) (fig. S4D, blue), consistent with our 16*S* ribosomal RNA sequencing (rRNA-seq) results (fig. S2). Additional contributors included mucin-degrading *Akkermansia* (*22*) (figs. S3C and S4E, green) and simpler carbohydrate-metabolizing *Lactobacillus* (*23*, *24*) (figs. S3D and S4G, yellow). Collectively, these results comport with the notion that WF enriched the carbohydrate catabolizing capacity of the microbiota, potentially in a manner related to the protection against inflammation it conferred.

To assess microbiota’s contribution to mediating WF’s impacts, we first used broad-spectrum antibiotics (fig. S5A). We found no difference in the severity of DSS-induced colitis between LF-fed and WF-fed mice under antibiotic treatment, suggesting that microbiota mediated WF’s protection in this model. To investigate the role of some of the specific taxonomic changes that resulted from the WF diet, we turned to gnotobiotic mice, specifically mice colonized with the eight-species bacteria consortium known as altered Schaedler flora (ASF) (*25*), to which *B. theta* and *A. muc* were added (Fig. 2G and fig. S5B). As the differences in response to DSS colitis between GBC-fed and LF-fed mice (both in conventional and ASF) have been well described in previous studies (*6*, *7*), we focused on the differences between LF-fed and WF-fed mice and, to aid logistics, relied on morphologic and histopathologic colitis readouts; WF failed to protect ASF mice against DSS colitis. However, colonizing ASF mice with *B. theta*, and to a lesser extent *A. muc*, restored WF-induced protection in this colitis model. Moreover, unlike *A. muc*, whose abundance is enriched in the absence of dietary fiber, as observed previously (*26*), the abundance of *B. theta* was positively correlated with the abundance/availability of dietary fiber (Fig. 2, E and F). These results strengthen the notion that WF-induced changes in microbiota contribute to its beneficial impacts and indicate that *B. theta* is one bacterium capable of mediating WF’s benefits. Furthermore, they indicated that ASF mice ± *B. theta* could serve as a tool for reductionist mechanistic study.

### WF’s protection against colitis associated with alterations in intestinal macrophages

**Fig. 3.**
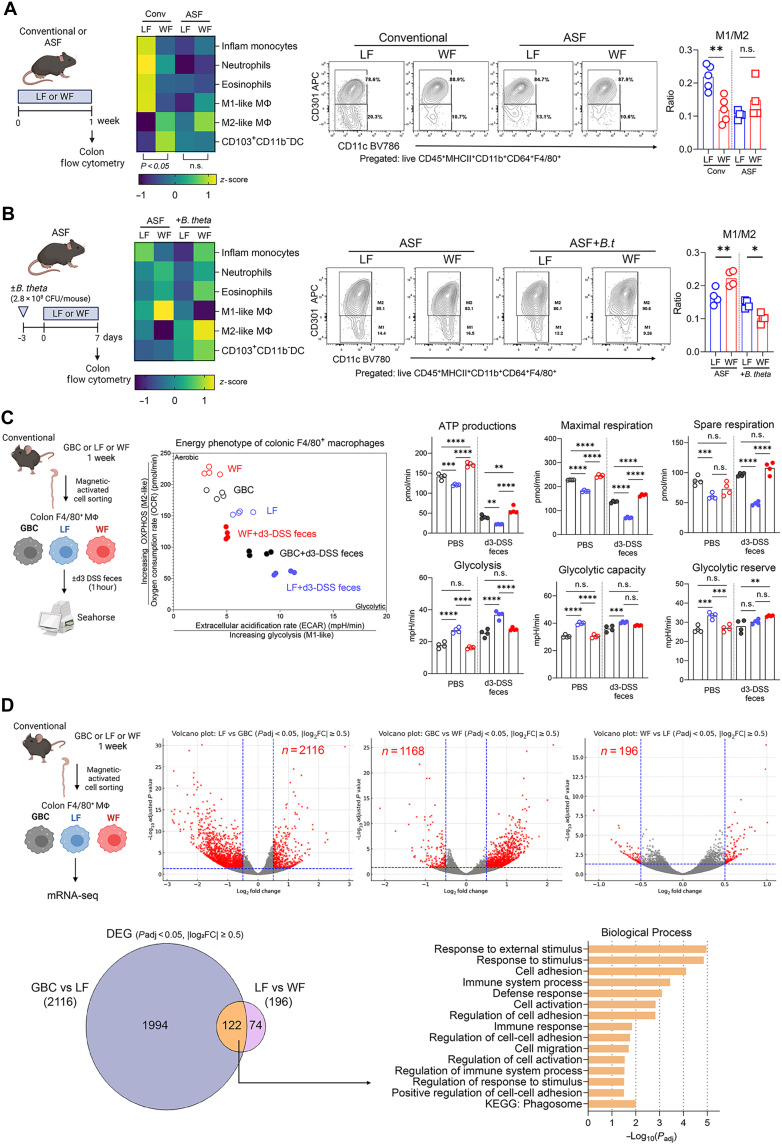
*B. theta* restored the WF-associated, microbiota-dependent macrophage phenotype in the colon. (**A**) B6 conventional (*n* = 5 per group) and ASF mice (*n* = 4 per group) were fed either LF or WF for 1 week. The lamina propria of the colon was analyzed by flow cytometry to assess innate immune cells and the M1/M2 macrophage ratio. (**B**) ASF mice (*n* = 4 per group) with or without *B. theta* were fed either LF or WF for 1 week. Flow plots for (A) and (B) are in figs. S6 to S8. (**C**) B6 conventional (*n* = 4 per group) mice were fed with GBC, LF, or WF for 1 week, and colon F4/80+ macrophages were isolated by magnetic beads. After 1-hour incubation in the presence or absence of fecal matter from day 3 (d3) DSS-treated mice, cellular metabolism was analyzed by Seahorse. Basal energy phenotype including OCR and ECAR were measured, as well as mitochondrial respiration and glycolysis. Real-time OCR and ECAR results are in fig. S9. (**D**) B6 conventional (*n* = 4 per group) mice were fed GBC, LF, or WF for 1 week. Colon F4/80^+^ macrophages were isolated using magnetic beads, and their transcriptomes were analyzed by mRNA-seq: volcano plots from DESeq2 results and GO enrichment analysis on DEGs. The results are representative of three independent experiments. All data are presented as mean values ± SEM. Statistical evaluation was conducted by one-way ANOVA (A: *P* = 0.0024; B: *P* < 0.0001) followed by Tukey’s multiple comparisons test (A and B) or Sidak’s multiple comparisons test (C). n.s. *P* > 0.05; *P < 0.05; **P < 0.01; ***P < 0.001; ****P < 0.0001. Schematics were created with BioRender.com. Gewirtz, A. (2026) https://BioRender.com/l3rbap9.

To gain a better understanding of how WF protected mice against colitis, we next turned to gene expression profiling. We reasoned that any differences seen following DSS treatment would largely reflect reduced inflammation and thus focused on WF’s impact on gene expression in the absence of DSS. Specifically, we performed RNA sequencing (RNA-seq) on total colon mRNA from ASF mice colonized, or not, with *B. theta* and fed the LF or WF diet (fig. S5C). A relatively small number of genes exhibited significant WF-induced changes in a *B. theta*–dependent manner, and those changes did not provide obvious clues into WF’s mechanism of action. Hence, we next used flow cytometry to profile intestinal innate immune cells, which are pivotal mediators of intestinal inflammation. We first assessed the impact of WF on colonic immune cellularity in conventional and ASF mice (Fig. 3A and figs. S6 and S7). In conventional mice, WF feeding decreased the relative and absolute abundance of several “pro-inflammatory” leukocytes, including M1-like macrophages, inflammatory monocytes, neutrophils, and eosinophils to levels similar to those of GBC-fed mice. Moreover, WF resulted in increased abundance of anti-inflammatory cells, including CD103^+^CD11b^−^ dendritic cells (DCs) and M2-like macrophages, whereas levels of total DCs did not change. Consequently, WF reduced the M1/M2 macrophage ratio, which is thought to broadly reflect inflammatory tone. None of these WF-induced changes in innate immune cells were observed in ASF mice, suggesting that they might contribute to protection against colitis.

Given that colonization of ASF mice with *B. theta* restored WF’s ability to ameliorate colitis severity, we reasoned that it should also restore any WF-induced changes in innate immune cells that were critical mediators of such protection. Hence, we analyzed intestinal immune cells in LF-fed and WF-fed ASF mice, colonized or not with *B. theta* (Fig. 3B and fig. S8). *B. theta* restored WF-induced changes in intestinal macrophages, but not other innate cells, that we had observed in conventional mice, suggesting that these cells are potential key mediators of WF’s ability to ameliorate colitis. Reexamining our total colon mRNA sequencing (mRNA-seq) data with appreciation of a possible role for intestinal macrophage polarization led us to note, and verify by PCR (fig. S5C), increased expression of *Ctrp3*, an anti-inflammatory adipokine that drives M2 macrophages in vitro (*27*) and reduces the severity of DSS colitis when overexpressed in the colons of transgenic mice (*28*).

We next sought to examine how WF feeding altered metabolism of colon macrophages, basally and in response to inflammatory challenge, specifically a challenge that could be uniformly applied to these cells without influences from other colon cells. Hence, we isolated intestinal macrophages from conventional mice fed GBC, LF, or WF diets and subjected them to real-time metabolic analysis (i.e., Seahorse) basally or following exposure to feces or colon supernatant from DSS-treated GBC-fed mice. Colonic macrophages from WF-fed mice exhibited increased oxygen consumption rates (OCRs), reflecting enhanced mitochondrial respiration and oxidative phosphorylation (OXPHOS). In contrast, macrophages from LF-fed mice demonstrated heightened extracellular acidification rates (ECARs), indicating a greater reliance on glycolytic metabolism, rather than OXPHOS, to generate energy. Following stimulation with feces or colon supernatant collected from day 3 of DSS-treated mice, macrophages from WF-fed mice maintained relatively high OCR and displayed only a moderate shift toward ECAR (Fig. 3C and fig. S9). This metabolic phenotype is associated with anti-inflammatory M2 macrophage polarization, which plays a crucial role during intestinal inflammation by promoting tissue repair, wound healing, and resolution of inflammation (*29*). Conversely, colonic macrophages from LF-fed mice exhibited a significant shift toward enhanced glycolysis and reduced OXPHOS activity, a hallmark of pro-inflammatory M1-like macrophages that exacerbate tissue damage during intestinal inflammation.

Application of mRNA-seq to these colonic macrophages revealed that switching the host’s diet from GBC to LF resulted in 2116 differentially expressed genes (DEGs) (Fig. 3D). The switch to WF had a comparatively moderate impact, 1168 DEGs, whereas comparing WF to LF yielded only 196 DEGs, of which 122 restored gene expression toward GBC. Accordingly, principal components analysis (PCA) indicated that WF only partially restored changes in colon macrophage gene expression induced by LF (fig. S10A). The difference between WF and GBC potentially reflected that GBC differed from the LF diet in many ways besides fiber and/or that WF by itself could not fully recapitulate the impacts of the complex fiber mixtures in GBC. Regardless, that WF protected mice against DSS colitis suggested the changes in colonic macrophage gene expression resulting specifically from enriching the LF diet with WF might provide mechanistic insights. Analyzing the 122 WF-restored genes by gene ontology (GO) enrichment analysis found that WF had affected immune responses, cellular response to stimuli/stress, and cell adhesion pathways (*30*–*32*), all of which are related to M1/M2 macrophage polarization. GO enrichment analysis on DEGs without considering fold change identified additional biological processes, such as cellular metabolism/macromolecule catabolism, chemotaxis, and wound healing (fig. S10B), which also directly distinguish M1-like or M2-like macrophages. Collectively, these data suggest that WF consumption can rewire colonic macrophage metabolism and transcription to favor resolution of inflammation and mucosal healing.

### WF-derived metabolite training of macrophages dampened inflammation

**Fig. 4.**
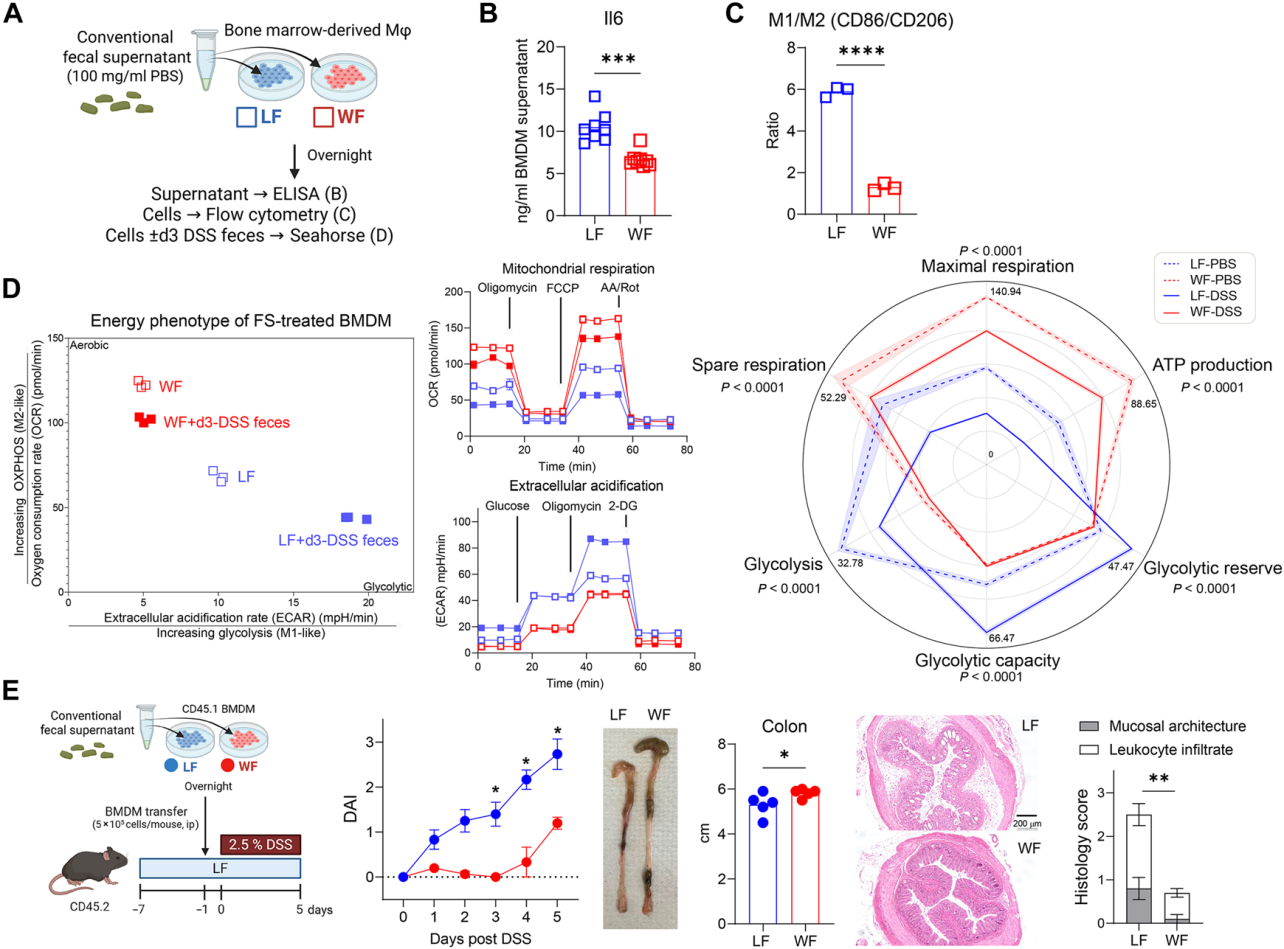
WF-derived metabolites trained BMDMs to mediate colitis protection. (**A**) Schematic for (B), (C), and (D). BMDMs were treated with LF or WF FS (100 mg/ml, 1:50 dilution) in the presence of LPS (10 ng/ml) overnight. (**B**) Il6 from the BMDM supernatant was quantified by ELISA (*n* = 8 per group). (**C**) M1-like and M2-like macrophages were quantified by flow cytometry (*n* = 3 per group). (**D**) BMDM metabolism was analyzed by Seahorse after 1-hour incubation in the presence or absence of feces from day 3 DSS mouse (*n* = 3 per group). AA/Rot, antimycin A/rotenone; 2-DG, 2-deoxy-d-glucose. (**E**) CD45.2 mice (*n* = 4 per group) were fed with LF for 1 week and given DSS water for 5 days. One day before DSS treatment, LF or WF FS-treated CD45.1 BMDMs (5 × 10^5^ cells per mouse) were intraperitoneally (ip) injected. Weight loss, DAI, colon length, and histology score were assessed. The results are representative of three independent experiments. All data are presented as mean values ± SEM. Statistical evaluation was conducted by the unpaired two-tailed *t* test (B: *P* = 0.0001; C: *P* < 0.0001;) one-way ANOVA (D) or two-way ANOVA (E) followed by Sidak’s multiple comparisons test. n.s. *P* > 0.05; *P < 0.05; **P < 0.01; ***P < 0.001; ****P < 0.0001. Schematics were created with BioRender.com. Gewirtz, A. (2026) https://BioRender.com/v3lkzro.

Although WF preserved *B. theta* levels in conventional mice, it did not affect the level of *B. theta* colonization when it was administered to ASF mice (Fig. 2G). Yet, it was the combination of WF and *B. theta* that protected ASF mice against colitis and changed macrophage phenotype, leading us to hypothesize that a WF–*B. theta* interaction had generated soluble mediators that affected macrophage polarity. To investigate this possibility, we applied fecal supernatants (FSs) to bone marrow–derived macrophages (BMDMs). BMDMs were exposed to FSs from conventional mice fed the LF or WF diets overnight, and the supernatant and cells were analyzed (Fig. 4A and fig. S11A). WF FS changed the Il6 secretion (Fig. 4B), M1/M2 ratio (Fig. 4C), and the cellular metabolism (Fig. 4D) of BMDMs. The pattern of results was reminiscent of what was observed in colon macrophages in vivo (Fig. 3C). Specifically, compared to FSs from LF-fed mice, WF FSs led to decreased glycolysis and greater maximal and spare respiration capacity.

Next, we examined the extent to which these macrophages, whose metabolism was altered by FSs in a diet-specific manner in vitro, might have the capacity to influence inflammation in vivo. Accordingly, FS-exposed BMDMs were adoptively transferred into LF-fed mice (5 × 10^5^ cells per mouse, intraperitoneally), which were then subjected to DSS challenge (Fig. 4E). Recipients of WF FS-trained BMDMs exhibited less severe colitis than recipients of BMDMs exposed to LF FSs with a particularly stark difference in disease activity index (DAI) score and histology score. Postmortem analysis of the colon by flow cytometry (fig. S11B) found that 10% of the WF FS-exposed cells (CD45.1), which could be distinguished from endogenous cells (CD45.2) by their congenic CD45 marker, could be recovered from the colon, indicating that a significant portion of these cells had reached this organ and, moreover, persisted through DSS challenge. A smaller portion of the LF FS-exposed cells was recovered following DSS colitis, potentially reflecting an intrinsic difference in their capacity to survive and/or the more severe inflammation exhibited by their host. Collectively, these results indicate that microbiota-mediated metabolism of WF generates metabolites that polarize macrophages toward an M2-like phenotype that mitigates the severity of colitis.

### Isofraxidin, a WF-derived metabolite, trained macrophages and dampened inflammation

**Fig. 5.**
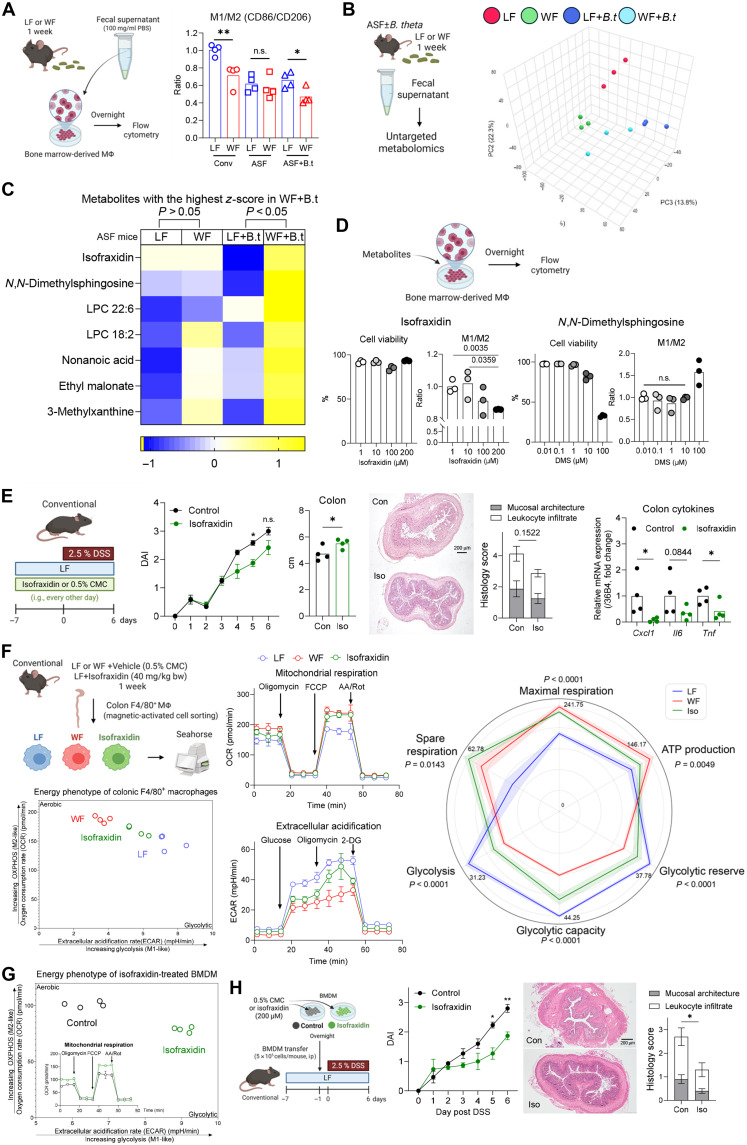
Isofraxidin recapitulates WF-derived macrophage-mediated colitis protection. (**A**) BMDMs were treated overnight with LF FS or WF FS (100 mg/ml), and M1/M2 ratio was analyzed by flow cytometry. (**B**) FSs of ASF mice (*n* = 4 per group) fed either LF or WF and colonized with or without *B. theta* were analyzed by untargeted metabolomics and the PCoA plot. (**C**) Seven candidates significantly increased by WF only in the presence of *B. theta* and had the highest average *z*-score in the WF+B.t group. LPC, lysophosphatidylcholine. (**D**) M1-like and M2-like macrophages were quantified by flow cytometry from BMDMs treated with either isofraxidin or DMS overnight in the presence of LPS (10 ng/ml). (**E**) Conventional mice (*n* = 4 per group) were gavaged with isofraxidin (40 mg/kg body weight) or 0.5% carboxymethyl cellulose (CMC) while on LF and then challenged with 2.5% DSS water for 6 days. i.g., intragastric. (**F**) Conventional mice (*n* = 4 per group) were gavaged with isofraxidin [40 mg/kg body weight (bw)] on LF or 0.5% CMC while on LF or WF. Then, colon F4/80^+^ macrophages were isolated using beads and analyzed by Seahorse. See fig. S14 for details. (**G**) BMDMs were treated overnight either with isofraxidin (200 μM) or 0.5% CMC and were analyzed by Seahorse. See fig. S14 for details. (**H**) Conventional mice (*n* = 5 per group) were fed with LF for 1 week and given DSS water for 5 days. One day before DSS treatment, isofraxidin- or vehicle-treated BMDMs (5 × 10^5^ cells per mouse) were intraperitoneally injected. DAI and histology score were assessed. The results are representative of three independent experiments. All data are presented as mean values ± SEM. Statistical significance was assessed using one-way ANOVA followed by Sidak’s multiple comparisons test (A, D, and F), two-way ANOVA followed by Sidak’s multiple comparisons test (DAI for E and H), or the unpaired two-tailed *t* test (C, E, and H). n.s. *P* > 0.05; *P < 0.05; **P < 0.01. Schematics were created with BioRender.com. Gewirtz, A. (2026) https://BioRender.com/lgypic8.

The results above led us to hypothesize that metabolism of WF by *B. theta* produced metabolites capable of promoting an M2 macrophage phenotype. To investigate this possibility, we applied FSs from different mice fed LF or WF to BMDMs (Fig. 5A). FSs from WF-fed conventional and ASF/*B. theta* mice, but not ASF FSs, reduced the ratio of M1/M2 expression markers (CD86/CD206), supporting our hypothesis. Culture media from *B. theta* grown in the presence of WF did not affect BMDM polarity, suggesting that ASF bacteria and/or host metabolism contribute to the ability of FSs to affect these cells (fig. S11C).

Untargeted liquid chromatography–mass spectrometry (LC-MS) metabolomics analysis indicated that WF, *B. theta*, and their combination affected the fecal metabolome (Fig. 5B). We specifically sought to define metabolites that were significantly increased by WF in the presence, but not in the absence, of *B. theta*. Of the 1052 defined metabolites detected across all the samples, only 9 were significantly elevated in FSs from ASF mice administered both WF and *B. theta* relative to those administered LF and *B. theta* either or neither, and 7 remained (Fig. 5C) after excluding the ones that are hydrophilic without any known transporters as they can hardly be diffused into the colon lamina propria to affect macrophages (fig. S12). Although any of these putative metabolites could have conceivably contributed to WF’s impacts, two of them were tested for further study because they were most significantly increased and, furthermore, their induction absolutely required both WF and *B. theta*, whereas the rest were modestly elevated by either WF or *B. theta* alone.

One such candidate metabolite was isofraxidin, which is a polyphenol, a class of bioactive plant-derived molecules plausibly present in WF, and, furthermore, has anti-inflammatory activity (*33*). The other candidate metabolite further studied was a sphingolipid derivative [*N*,*N*-dimethylsphingosine (DMS)], which was particularly intriguing in that sphingolipids can be produced by *B. theta*, and subsequently incorporated into host sphingolipid synthesis pathways (*34*) and, furthermore, may prevent *B. theta* from promoting inflammation (*35*). Testing these candidates in the BMDM polarization assay found that isofraxidin, but not DMS, lowered the M1/M2 ratio without reducing viability (Fig. 5D).

To test the potential significance of this result in vivo, LF-fed conventional mice were administered isofraxidin (40 mg/kg body weight), by oral gavage every other day for 1 week before, and during, DSS challenge. Analogous to previous findings in GBC-fed mice (*33*), this treatment protected against DSS colitis, albeit not as strongly as WF (Fig. 5E). In further accord with the in vitro BMDM assay, administration of DMS to mice (200 μg, 1 mg, or 4 mg/kg body weight, intraperitoneally) did not protect mice against DSS colitis (fig. S13A). However, a *B. theta* strain engineered to lack serine palmitoyltransferase (SPT), and thus unable to produce sphingolipids (*34*), conferred only a modest level of WF-induced protection against DSS colitis, suggesting that DMS may play an indirect role in WF’s mitigation of colitis (fig. S13B).

That isofraxidin protected mice against colitis and polarized BMDMs into M2-like macrophages led us to probe whether isofraxidin’s protection against colitis was mediated by macrophages. Isofraxidin shifted colonic macrophages’ basal (i.e., without DSS) metabolism toward an M2-like, WF-like phenotype (Fig. 5F and fig. S14A). This isofraxidin-derived metabolic phenotype was also recapitulated in vitro, wherein BMDMs were treated with isofraxidin (200 μM, overnight) (Fig. 5G and fig. S14B). Last, transplant of isofraxidin-treated BMDMs (5 × 10^5^ cells per mouse) into mice before DSS challenge reduced colitis severity (Fig. 5H). Thus, WF and *B. theta* interaction generated fecal metabolite, isofraxidin, which altered the cellular metabolism of colonic macrophages in a manner that protected against colitis.

## DISCUSSION

Broad societal changes in food production and dietary habits have resulted in reduced levels of per person fiber consumption. Reduced fiber intake, within and across societies, is associated with increased risk for a broad array of chronic inflammatory diseases. These observations, combined with appreciation that fiber directly interacts with gut microbiota, which, itself, has a broad influence on health, have led to the widespread belief that reduced fiber consumption has contributed to the increased prevalence of such diseases and, consequently, that enriching foods with semipurified plant fibers could prevent them. Our investigation of this hypothesis over the past few years has led us to appreciate that fiber, defined as the components of plant-based foods that are not catabolized by the enzymes of their consumer, encompasses a highly diverse group of molecules, all carbohydrate-based yet containing a broad range of bioactive chemical motifs. Fibers isolated from distinct plants have markedly distinct impacts on gut microbiota and, consequently, host health, although such findings largely focused on fibers derived from plants not widely consumed in the West. Accordingly, the goal of this study was to study the impact of a specific fiber type, namely, WF, a once ubiquitous component of the Western diet that became much less widely consumed as food production evolved. We observed that enrichment of a highly refined LF diet with WF mitigated the loss of bacterial diversity that otherwise resulted from switching mice from GBC, a diet naturally rich in fiber, to an LF diet. Furthermore, WF feeding resulted in polarizing intestinal macrophages toward an M2-like phenotype and reduced severity of DSS-induced colitis. These findings support the hypothesis that the widespread adoption of bran removal in generating wheat-based foods has contributed to increased incidence of chronic inflammatory diseases. Furthermore, they suggest that incorporating WF into processed foods may make them healthier.

Although polarization of immune cells toward an anti-inflammatory phenotype is a well-appreciated consequence of some specific dietary fibers, the mechanisms by which WF dampens inflammation were distinct from those characterized for fiber supplements, namely, inulin and psyllium, in that it involved neither SCFAs nor the FXR bile acid receptor. Rather, WF-feeding polarized intestinal macrophages toward an M2-like phenotype via soluble metabolites that required both WF and *B. theta*. Characterization of such metabolites via untargeted metabolomics suggests a role for molecules that are synthesized and/or possibly liberated from WF by *B. theta* including, respectively, sphingolipids and isofraxidin.

Our working hypothesis is that microbiota CAZymes “liberate” isofraxidin, or a precursor, from WF. Isofraxidin is not a widely recognized component of whole wheat, but most chemical analyses of wheat have generally focused on its carbohydrates, rather than polyphenolic compounds. Nonetheless, it is appreciated that WF contain various phenolic compounds, and our linkage analysis also detected nonspecific “noncarbohydrate” bonds (FS 1C). Even after purification, a significant portion of polyphenols can remain strongly attached to fibers, for instance, through ester bonds. Ferulic acid–esterified arabinoxylan is one such case, where feruloyl esterase from *Bacteroides intestinalis* can hydrolyze the ester bond between ferulic acid and the arabinosyl residue (*36*, *37*). Isofraxidin, a coumarin predominantly found in ginseng or medicinal herb *Acanthopanax senticosus* (*38*, *39*), is not thought to be a common grain-derived polyphenol. However, coumarins can be converted from ferulic acids by plant enzymes (*40*), suggesting the possibility that small amounts of these coumarins could be attached to a WF. Another potential source of isofraxidin is its formation from the precursor fraxetin, which was also detected by untargeted metabolomics data with a lower abundance in the WF+*B.t* group compared to the other groups. The conversion of fraxetin to isofraxidin can be mediated by coumarin methyltransferase, which has been characterized more extensively in plants than in bacteria or mammals (*40*), whereas hosts (humans and mice) and bacteria do express various *O*-methyltransferases, including some that act on coumarins.

We do not anticipate that isofraxidin generation is dependent on *B. theta* sphingolipid generation but cannot rule out this possibility. We do not know the relative role of either sphingolipids or isofraxidin, but we assume that the levels generated in vivo contribute to, but are not sufficient to fully convey, benefits of WF. We also do not know the relative role of *B. theta* in conferring benefits of WF in conventional mice. Rather, we envisage that the liberation of phytochemicals from WF by bacteria rich in CAZymes, including *B. theta*, as being a general paradigm that mediates benefits of some fibers as commensal in both humans and mice. Perhaps the best molecularly characterized example of this concept is the pioneering findings by Pereira *et al.* (*36*) that a select gene locus in a Bacteroidetes species was able to degrade arabinoxylans to liberate ferulic acid, which is also reported to have anti-inflammatory activity. Polysaccharide-incarcerated phytochemicals whose liberation requires gut bacteria not only clearly meet the definition of dietary fiber but also highlight that this is an extremely broad nutrient class that has potential to affect host health far beyond merely providing bulk and serving as a carbon source for microbiota. How liberated phytochemical impact inflammation will likely be quite heterogeneous but, in the case of isofraxidin, may involve it binding to the TLR4/MD-2 complex and subsequently impeding lipopolysaccharide (LPS)–induced TLR4 signaling (*41*).

Although this study focused on the DSS model of acute colitis, largely due to its tractability, we anticipate that the results would be generally applicable to other models of intestinal inflammation. Although benefits of WF in the DSS model could be attributed to macrophages, other intestinal immune cells were nonetheless affected, as observed in our parallel study where WF-promoted regulatory T cells protected mice against T cell–mediated colitis (*42*). Extrapolating results of our mouse diet studies to humans is inherently difficult. This caveat notwithstanding, although we have no reason to believe that WF would benefit individuals with established IBD, we speculate that its consumption may reduce risk of developing this disorder.

Increasing WF consumption can, of course, be achieved by eating foods made from whole wheat flour. Another means of WF consumption is incorporating semipurified versions of it into shelf-stable processed foods. The specific WF used herein is commercially produced and supplied to food companies for this purpose. Preference for one of these approaches is likely not specific for WF per se but likely broadly extends to the extent to which one believes highly processed foods should be discouraged versus reformulated. We submit that a better understanding of how fiber interacts with the microbiota to influence host health is germane to either approach and that the findings herein represent a significant step toward this goal.

## MATERIALS AND METHODS

### Mice

Male and female C57BL/6 wild-type (WT) were purchased from the Jackson Laboratory (Bar Harbor, ME). Gnotobiotic mice with ASF were generated from C57BL/6 germ-free mice purchased from Taconic Biosciences (Rensselaer, NY). All mice were bred/housed and/or studied at Georgia State University under the Institutional Animal Care and Use Committee (#A20043 and #A24001). ASF mice were studied in isocages to prevent contamination. Initial experiments (Figs. 1 to 3; comparing WF’s DSS responses including ASF mice) were performed using both male and female mice, but no sex-specific differences were observed. Consequently, female mice were used for all subsequent experiments. All mice were maintained on grain-based rodent chow from Lab diets (catalog no. 5001) except where indicated otherwise.

### Diets

The composition of the defined diets used herein is outlined in Table 1. These diets were all generated by Research Diets Inc. (New Brunswick, NJ) using their standard ingredients except for Vitacel WF, minimally or highly refined oat fiber (Oat^min^ and Oat^hi^), which were provided by JRS Inc. (Rosenberg, Germany). For all experiments with gnotobiotic mice, these diets were γ-irradiated diets as previously described (*5*). Unless otherwise indicated, the switch to GBC to the indicated diet was initiated at 6 to 8 weeks of age. Hops (*Humulus lupulus* L.) beta acids were provided by M. Flythe (USDA) and given to mice in drinking water (40 parts per million). For antibiotic treatment, mice were administered drinking water with ampicillin (1 g/liter), neomycin (1 g/liter), and metronidazole (1 g/liter), which were replaced every 3 to 4 days. Metabolite administration was done as previously described (*33*, *41*, *43*); mice were orally gavaged with isofraxidin (Sigma-Aldrich, PHL89229) (40 mg/kg body weight) or 0.5% carboxymethyl cellulose or intraperitoneally injected with DMS (Sigma-Aldrich, SML0311) (0.2, 1, or 4 mg/kg body weight) or vehicle [phosphate-buffered saline (PBS)] every other day.

### WF complex carbohydrate composition analysis

The wheat composition of JRS Vitacel WF was analyzed by the Complex Carbohydrates Research Center at the University of Georgia as previously described (*44*, *45*). The major carbohydrates in WF were determined by analyzing the glycosyl composition and linkage residue. The analysis was performed by combined gas chromatography–mass spectrometry (GC-MS) of the *O*-trimethylsilyl (TMS) methyl glycoside derivatives produced from the sample by acidic methanolysis.

### DSS-induced colitis

Six- to 8-week-old female mice were fed with designated diets for 1 week, and mice were administered drinking water containing 2.5% DSS (MP Biomedicals, #S7102; molecular weight: 36,000 to 50,000) while maintaining the diets. Mice were monitored daily for body weight, stool consistency, and blood in feces to score the DAI (see Table 2 for criteria) until the end of the experiment. Blood in feces was analyzed upon collection using the Hemoccult single-slide rapid diagnostic test kit (Hemocue, Ängelholm, Sweden). Mice were euthanized when one or more mice reached 20% weight loss compared to the weight on the initial DSS administration (*46*). Colon length and spleen weight were measured, and tissues and feces were collected for downstream analysis.

**Table 2.** Criteria for DAI scoring.

Score	Weight loss	Stool consistency	Blood in feces
0	No loss or gain	Normal	Negative hemoccult test
1	>0% loss of body weight	Soft stool	–
2	>5%	Loose/watery diarrhea	Positive hemoccult test
3	>10%	Slimy diarrhea with blood	–
4	>15% loss of body weight	Severe diarrhea with blood	Visible blood

### *B. thetaiotaomicron* culture and administration

*B. thetaiotaomicron* [American Type Culture Collection (ATCC) 29148, VA], herein referred to as *B. theta*, was initially activated in anaerobic chopped meat medium (ATCC medium 1490) for 48 hours, and several drops were spread on the sheep blood agar plate. Then, a single colony was picked and cultured in brain heart infusion (BHI) medium (Sigma-Aldrich) for 24 to 48 hours at 37°C under the anaerobic chamber (AS-580, Anaerobic Systems, CA) (5% carbon dioxide, 5% hydrogen, and 90% nitrogen). *B. theta* sphingolipid-deficient bacteria were generated as previously described (*34*). *B. theta* WT or sphingolipid-deficient bacteria were given to ASF mice [2 × 10^8^∼3 × 10^8^ colony-forming units (CFU) per mouse; confirmed by plate counting] 3 days before diet intervention.

### Isolation of intestinal lamina propria

Dissected small and large intestines were opened longitudinally and cut into small pieces and washed with calcium-free and magnesium-free Hanks’ balanced salt solution (CMF/HBSS). The epithelium was removed by two consecutive incubations in a shaker (200 rpm, 20 min, 37°C) with 2 mM EDTA in CMF/HBSS containing 5% fetal bovine serum. Intestinal tissue pieces were digested with collagenase type IV (1 mg/ml; Sigma-Aldrich, C5138) and DNase I (40 μg/ml; Roche, Germany) in CMF/HBSS or RPMI containing 5% fetal bovine serum at 37°C for 10 min. Cells were then filtered (40 μm), and supernatants were discarded and resuspended in CMF/HBSS. Lamina propria mononuclear cells were further purified by Percoll (GE Health Care, Uppsala, Sweden) density gradient centrifugation.

### Flow cytometry

Flow cytometry analysis was performed as described previously (*47*). Prepared cells were first blocked with 2.4G2 (BioXCell) to prevent Fc receptor–mediated binding. Live cells were identified by excluding dead cells using the Fixable Aqua Dead Cell Staining Kit (Invitrogen). After washing with PBS, cells were stained for surface markers using conjugated monoclonal antibodies (mAbs) in fluorescence-activated cell sorting (FACS) buffer. For intracellular staining, cells were fixed and permeabilized using the Fixation/Permeabilization (Fix/Perm) Buffer Set (eBioscience). Subsequently, cells were stained for CD206 in 1X Permeabilization Buffer (eBioscience). Fluorochrome-conjugated mAbs were used as indicated in the figures and Table 3. Multiparameter flow cytometry was performed on a CytoFlex (Beckman Coulter) and analyzed using FlowJo software (Tree Star).

**Table 3.** Antibodies for flow cytometry.

Antibody	Source	Identifier
Brilliant Violet 605 anti-mouse CD45	BioLegend	Catalog no. 103155; RRID: AB_2650656
Alexa Fluor 700 Rat Anti-Mouse Ly-6G	BD Biosciences	Catalog no. 561236; RRID: AB_10611860
PerCP-Cyanine5.5 Ly-6C mAb	eBioscience	Catalog no. 45-5932; RRID: AB_1518762
FITC anti-mouse/human CD11b	BioLegend	Catalog no. 101205; RRID: AB_312788
BD Horizon BV786 Hamster Anti-Mouse CD11c	BD Biosciences	Catalog no. 563786; RRID: AB_2738394
BD Horizon BV650 Rat Anti-Mouse I-A/I-E	BD Biosciences	Catalog no. 563415; RRID: AB_2738192
Brilliant Violet 421 anti-mouse CD64 (FcγRI)	BioLegend	Catalog no. 139309; RRID: AB_2562694
PE/Cyanine7 anti-mouse F4/80	BioLegend	Catalog no. 123114; RRID: AB_893478
APC anti-mouse CD301 (MGL1/MGL2)	BioLegend	Catalog no. 145708; RRID: AB_2562942
BD Horizon PE-CF594 Rat Anti-Mouse Siglec-F	BD Biosciences	Catalog no. 562757; RRID: AB_2687994
APC anti-mouse CD86	BioLegend	Catalog no. 105012; RRID: AB_493342
Alexa Fluor 700 anti-mouse CD206	BioLegend	Catalog no. 141734; RRID: AB_2629637
eBioscience CD45.1 mAb (A20), PE-Cyanine7	eBioscience	Catalog no. 25-0453-82; RRID: AB_469629
BD Pharmingen FITC Mouse Anti-Mouse CD45.2	BD Biosciences	Catalog no. 561874; RRID: AB_10894189

### BMDM culture and in vivo transfer

Bone marrow cells were isolated from WT female B6 mice and prepared according to Toda *et al.* (*47*). Approximately 5 × 10^5^∼6 × 10^5^ cells per well were spread in 96-well cell culture plates. On day 7, BMDMs were treated with FSs (100 mg/ml in PBS; 1:50 diluted in wells) or metabolites overnight in the presence of LPS (10 ng/ml). Metabolite concentrations for BMDM treatment were decided as previously described (*33*, *43*). Cells were collected the next morning and immediately stained for macrophages (CD86/CD206) for flow cytometry. CD45.1 BMDMs were prepared and treated overnight with FSs and intraperitoneally transferred (5 × 10^5^ cells per mouse) to CD45.2 mice to test BMDM-derived protection.

### RNA extraction and quantitative RT-PCR

Total RNA was extracted from colons using TRIzol (Invitrogen, Carlsbad, CA) according to the manufacturer’s protocol. Lithium chloride (Sigma-Aldrich, L7026) was used to purify the RNA of DSS-treated groups as described previously (*48*). Quantitative reverse transcription PCR (RT-PCR) was performed using the iTaq One-Step RT-PCR Kit with SYBR Green (Bio-Rad, Hercules, CA) in a CFX 96 apparatus (Bio-Rad, Hercules, CA). Differences in transcript levels were normalized to the housekeeping gene *36b4*. The primers used in this study are listed in Table 4.

**Table 4.** Primers for RT-qPCR.

Gene	Forward (5′-3′)	Reverse (5′-3′)
*36b4*	TCCAGGCTTTGGGCATCA	CTTTATTCAGCTGCACATCACTCAGA
*Cxcl1*	GCATTAGCTTCAGATTTACGGGT	CAGGGTCAAGGCAAGCCTC
*Il6*	TGGGGCTCTTCAAAAGCTCC	AGGAACTATCACCGGATCTTCAA
*Tnf*	GCCTCTTCTCATTCCTGCTTG	GCATTAGCTTCAGATTTACGGGT

### Histopathologic analysis

Fresh colons were fixed in 10% PBS-buffered formalin. Paraffin embedding, sectioning, and hematoxylin and eosin (H&E) staining were performed at HistoWiz Inc. High-resolution images of the stained slides were scored by histopathologists, blinded to the study protocol, assessing parameters such as inflammatory area, destruction of mucosal architecture, mucosal ulceration, cellular infiltrate, transmural inflammation, muscle thickening, crypt abscesses, and goblet cell depletion, as previously described (*7*).

### Cecal SCFA analysis

Cecal content was collected and then weighed before freezing. Levels of acetate, propionate, and butyrate in the samples were measured after extraction with ethyl acetate using an Agilent 7890A gas chromatograph (Santa Clara, CA) with a fused silica capillary column (Nukon SUPELCO no. 40369-03A, Bellefonte, PA) as previously described (*49*). Heptanoic acid is used as an internal standard, and the results are presented as μM/g of cecal contents.

### Lipocalin-2 ELISA

Fecal lipocalin-2 levels were measured using DuoSet mouse lipocalin-2 enzyme-linked immunosorbent assay (ELISA) kit (R&D Systems, #DY1857) according to the manufacturer’s protocol as previously described (*50*). FSs were collected after centrifugation (12,000 rpm, 10 min, 4°C) of the homogenized feces in PBS (100 mg/ml).

### Fecal bacterial DNA isolation and quantification by qPCR

Fresh fecal samples were collected before and after the diet intervention and stored at −20°C until analysis. Fecal bacterial DNA was extracted using the Qiagen QIAamp fast DNA stool mini kit (#51604). The total bacteria number was measured by qPCR using the QuantiNova SYBR Green PCR Kit (Qiagen, #208252) with 16*S* rRNA primers 8F: 5′-AGAGTTTGATCCTGGCTCAG-3′ and 338R: 5′-CTGCTGCCTCCCGTAGGAGT-3′. Results are expressed as bacteria number per mg of feces, using a standard curve (*7*). For quantification of *A. muciniphila* and *B. thetaiotaomicron* in feces, the following primers were used: *A. muc* forward: 5′-CAGCACGTGAAGGTGGGGAC-3′, *A. muc* reverse: 5′-CCTTGCGGTTGGCTTCAGAT-3′, *B. theta* forward: 5′-CCGAGCGTTATCCGGATTTAT-3′, and *B. theta* reverse: 5′-GCACCTTCACATTTGCCTTAC-3′.

### Macrophage metabolic analysis by Seahorse

The real-time metabolism of colon F4/80^+^ macrophages and FS-treated BMDMs was assessed using the Seahorse XF Cell Mito Stress Test and Glycolytic Stress Test Kits (Agilent Technologies) following the manufacturer’s protocol (*46*). Colon macrophages were isolated from GBC, LF, WF, or isofraxidin/vehicle-fed mice by beads. Sorted cells (5 × 10^4^ per well) were seeded in Seahorse plates and cultured overnight in RPMI 1640 medium containing 5% fetal bovine serum, 1% Hepes, 1% nonessential amino acids, 1 mM sodium pyruvate, 55 μM 2-mercaptoethanol, penicillin (100 U/ml), and streptomycin (100 μg/ml). The next day, cells were incubated in Seahorse assay medium for 1 hour with 2 mM glutamine (glycolysis test) or 10 mM glucose and 1 mM pyruvate (Mito Stress Test). Test reagents were resuspended in assay medium at final concentrations of 1.5 μM oligomycin, 4 μM carbonyl cyanide *p*-trifluoromethoxyphenylhydrazone (FCCP), and 0.5 μM rotenone/antimycin A (Mito Stress) or 10 mM glucose, 1 μM oligomycin, and 50 mM 2-deoxy-d-glucose (glycolysis stress). The Seahorse XF analyzer measured the OCR and ECAR. Glycolytic parameters were calculated as follows: basal glycolysis = pre-oligomycin ECAR − nonglycolytic ECAR; max glycolysis = ECAR post-oligomycin and pre-FCCP; glycolytic reserve = max − basal glycolysis. Mitochondrial respiration was determined as follows: basal respiration = pre-oligomycin OCR − nonmitochondrial OCR; max respiration = OCR post-FCCP and pre-rotenone/antimycin A.

### Gut microbiota profiling by 16*S* rRNA gene sequencing

Fecal DNA was extracted using the DNeasy 96 PowerSoil Pro QIAcube HT Kit (Qiagen), and the region V3-V4 of 16*S* rRNA genes was amplified using the following primers: 341F 5′-TCGTCGGCAGCGTCAGATGTGTATAAGAGACAGCCTACGGGNGGCWGCAG-3′; 805R 5′-GTCTCGTGGGCTCGGAGATGTGTATAAGAGACAGGACTACHVGGGTATCTAATCC-3′. PCR products of each sample were purified using Ampure XP magnetic purification beads. An index PCR was performed to attach dual barcodes and Illumina sequencing adapters using the Nextera XT Index kit (Illumina). Final PCR products were verified on 1.5% DNA agarose gel and quantified using Pico dsDNA assay (Invitrogen). An equal molar of each sample was combined and purified again using Ampure XP beads as the library. The library was diluted and spiked with 5% PhiX control (Illumina) and sequenced by an Illumina iSeq 100 sequencing system [2 × 150 base pairs (bp)]. Demultiplexed fastq files were generated on instrument. Sequence reads were quality filtered by the DADA2 plugin in Qiime2 (*51*). Taxonomy was assigned on the basis of the Greengenes database. Raw sequencing data have been deposited at the National Center for Biotechnology Information (NCBI) Sequence Read Archive (SRA) (PRJNA1249953). Phylogenetic Investigation of Communities by Reconstruction of Unobserved States 2 (PICRUSt2) analysis was performed to predict metagenomes from Qiime2 output files (taxonomy and representative sequence files) via the Galaxy server using the PICRUSt2 full pipeline. The functional profiles were summarized on the basis of EC numbers, and the relative contribution of taxa to each predicted enzyme was assessed.

### Untargeted metabolomics analysis (ultraperformance liquid chromatography–mass spectrometry)

Frozen fecal samples were sent to Creative Proteomics (Stony Brook NY) for supernatant preparation and untargeted metabolomics analysis. Separation was performed by ACQUITY UPLC (Waters) combined with Q Exactive MS (Thermo Fisher Scientific) and screened with electrospray ionization mass spectrometry (ESI-MS). The LC system is composed of ACQUITY UPLC HSS T3 (100 mm by 2.1 mm by 1.8 μm) with ACQUITY UPLC (Waters). The mobile phase is composed of solvent A (0.05% formic acid water) and solvent B (acetonitrile) with gradient elution. MS results from both ESI+ and ESI− modes were pooled together for analysis.

### mRNA-seq analysis

Total RNA from colon or bead-sorted colonic F4/80^+^ macrophages was extracted using the QIAGEN RNeasy Plus Mini Kit. The extracted RNA samples were sent to Novogene for library preparation and sequencing on the NovaSeq X Plus instrument using a high-output 2x 150-bp run. The mouse reference sequence file (GRCm39) and the annotated general feature format (gtf) file were downloaded from the Gencode website (https://gencodegenes.org/mouse/). The fastq files were used for sequencing read quality screening, and the RNA-seq data were analyzed using the Galaxy server following the RNA-seq tutorial at usegalaxy.org (https://training.galaxyproject.org/training-material/topics/transcriptomics/tutorials/ref-based/tutorial.html). Raw sequencing data have been deposited at the NCBI SRA [PRJNA1298791 (total colon from ASF mice) and PRJNA1298338 (colon macrophages from conventional mice)].

### Statistical analysis

GraphPad Prism 10.0 was used for all statistical testing. All data are given as means ± SEM and were compared via Student’s *t* test, analysis of variance (ANOVA) followed by Tukey’s multiple comparisons test, or multiple *t* tests, if certain comparisons were tested among more than three groups, followed by Bonferroni correction (alpha divided by the number of comparisons). Significance is expressed as *P < 0.05, **P < 0.01, ***P < 0.001, and ****P < 0.0001.

### Experimental schematics

All experimental schematics were created using BioRender (https://biorender.com/).
