# Supplementary material for: Wheat fiber mitigates colitis via non-SCFA microbial metabolite-trained intestinal macrophages
Source: Sci Adv. 2026 Mar 25;12(13):eaec5757. doi: 10.1126/sciadv.aec5757 (PMC13015863; doi:10.1126/sciadv.aec5757)
Supplement: Supplementary file 1 — Figs. S1 to S14 [file sciadv.aec5757_sm.pdf]

Science Advances
AAAS

Supplementary Materials for

Wheat fiber mitigates colitis via non-SCFA microbial metabolite-trained intestinal macrophages

Seong-eun G. Kim *et al.*

Corresponding author: Andrew T. Gewirtz, agewirtz@gsu.edu

Sci. Adv. **12**, eaec5757 (2026)
DOI: 10.1126/sciadv.aec5757

This PDF file includes:

Figs. S1 to S14

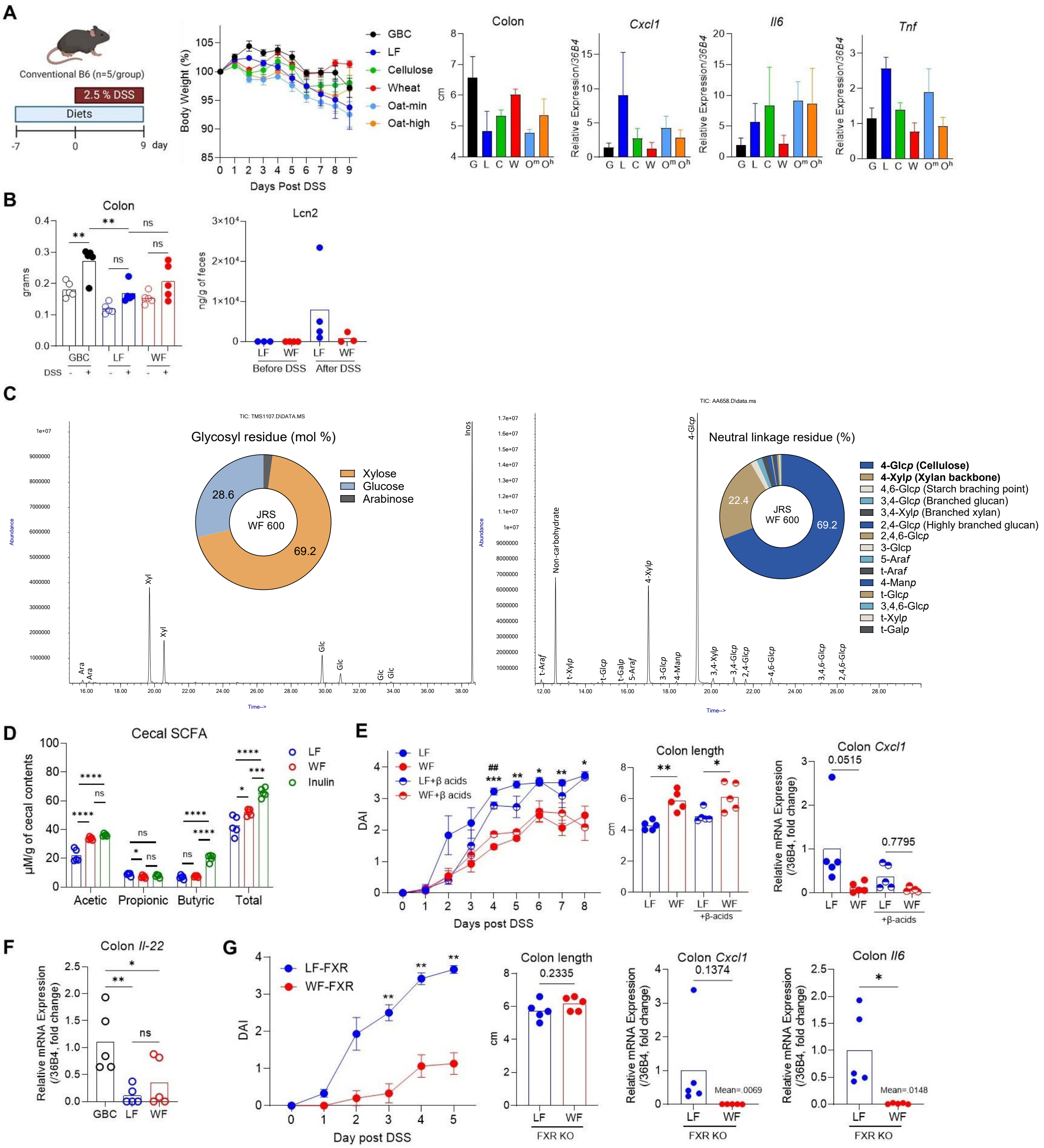

Supplementary Figure 1. WF-mediated colitis protection was not dependent on short-chain fatty acids, nor bile sensor FXR (Related to Figure 1).

(A) Seven-week-old male B6 mice (n=5 per group) were fed GBC, low fiber diet (LF), or LF-enriched with cellulose, wheat fiber (WF), Oat fiber-min, or Oat fiber-hi for one week, followed by 2.5 % DSS water for 9 days.

(B) Colon weight and fecal Lcn2 from Figure 1.

(C) Gas chromatogram of TMS methyl glycosides and glycosyl residue composition. And Gas chromatogram of the linkage residues (in mol %) and neutral linkage residue composition (in %). For the neutral linkages detected greater than 1%, the typical polymer source was annotated in parentheses.

Abbreviations for linkage residues: 4-Glcp, 4-O-substituted glucopyranosyl; 4-Xylp, 4-O-substituted xylopyranosyl; 4,6-Glcp, 4,6-di-O-substituted glucopyranosyl; 3,4-Glcp, 3,4-di-O-substituted glucopyranosyl; 3,4-Xylp, 3,4-di-O-substituted xylopyranosyl; 2,4-Glcp, 2,4-di-O-substituted glucopyranosyl; 2,4,6-Glcp, 2,4,6-tri-O-substituted glucopyranosyl; 3-Glcp, 3-O-substituted glucopyranosyl; 5-Araf, 5-O-substituted arabinofuranosyl; t-Araf, Terminal arabinofuranosyl; 4-Manp, 4-O-substituted mannopyranosyl, t-Glcp, Terminal glucopyranosyl; 3,4,6-Glcp, 3,4,6-tri-O-substituted glucopyranosyl; t-Xylp, Terminal xylopyranosyl; t-Galp, Terminal galactopyranosyl

(D) B6 mice (n=5/group) were fed LF, WF, or inulin diets for one week and cecal contents were analyzed for short-chain fatty acid (SCFA) concentrations, including acetic acid, propionic acid, and butyric acid, as well as total SCFAs.

(E) B6 mice (n=5/group) were fed either LF or WF for one week, followed by DSS water for 7 days. To inhibit intestinal bacterial fermentation, mice were given 40 ppm hops β acid in drinking water throughout the experiment. Body weight and feces were monitored for DAI, and colon length and colon *Cxcl1* were measured.

(F) GBC, LF, or WF-fed healthy mice were analyzed for colon Il22 transcript expression by qPCR.

(G) FXR KO mice (n=5/group) were fed either LF or WF for a week, and challenged with DSS water for 5 days. Body weight and feces were monitored for DAI, and colon length and colon *Cxcl1* and *Il6* were measured.

All data are presented as mean values ±SEM or individual values. Statistical significance was determined using one-way ANOVA with Tukey’s multiple comparison test (B, D, E, F) or two-way ANOVA followed by Šídák's multiple comparisons test (G) or unpaired two-way t test (G). ns P > 0.05, *P < 0.05, **P < 0.01, ***P < 0.001, ****P < 0.0001.

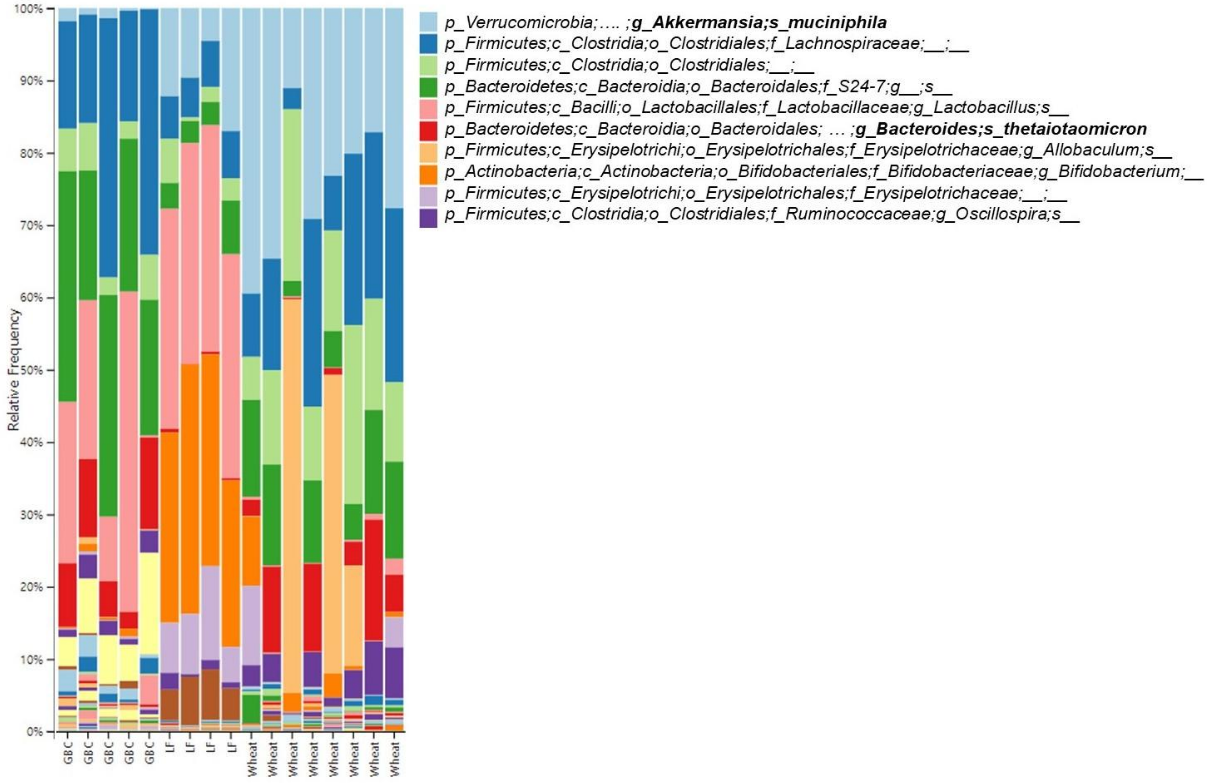

Supplementary Figure 2. WF altered microbiota composition at species level (Related to Figure 2).

B6 conventional mice (n=4-8/group) were fed GBC, LF, or WF for a week, and the feces were analyzed by 16S rRNA sequencing using iSeq. Taxonomy analysis at species level.

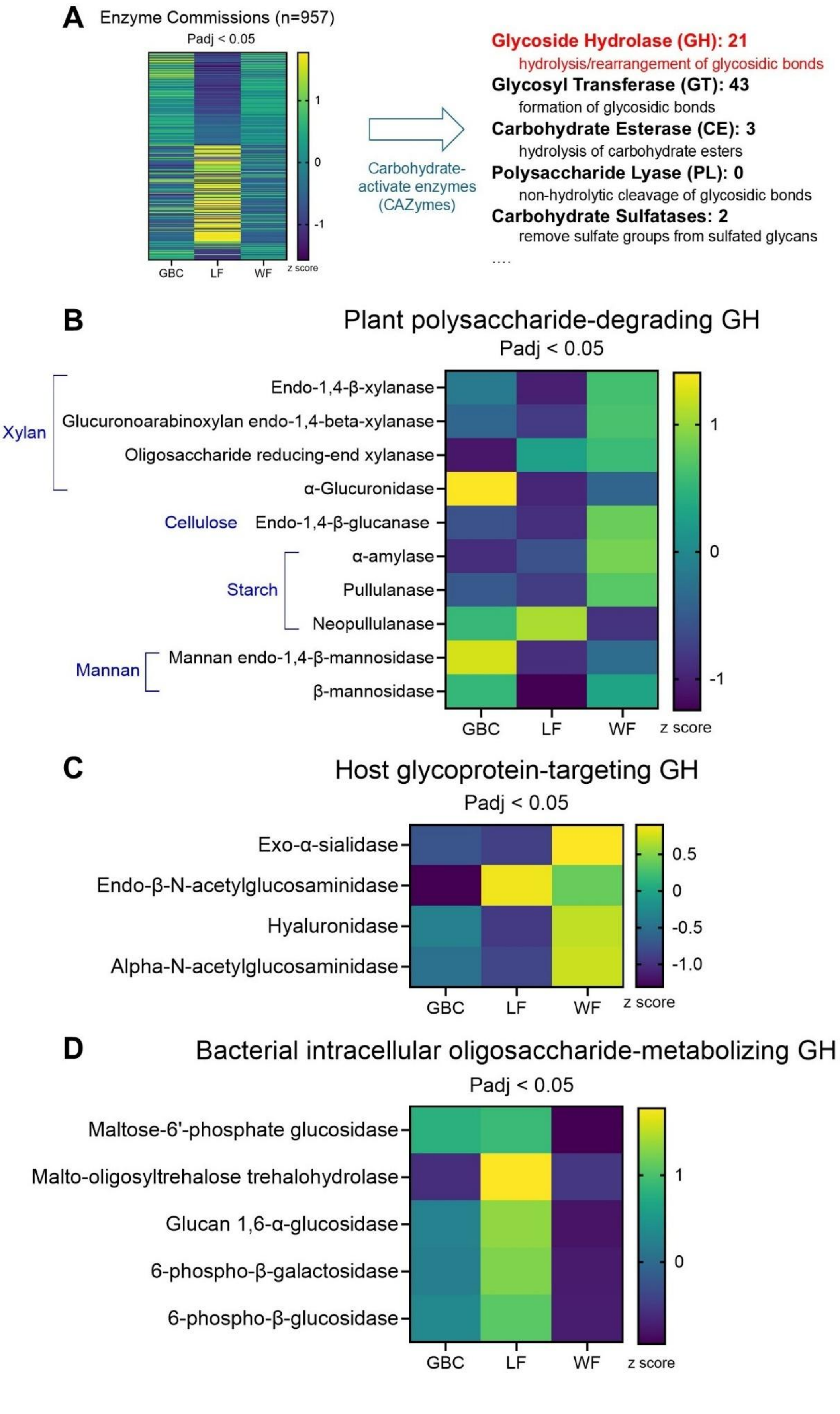

A
Enzyme Commissions (n=957)
Padj < 0.05
GBC
LF
WF
z score
Carbohydrate-activate enzymes (CAZymes)
Glycoside Hydrolase (GH): 21
hydrolysis/rearrangement of glycosidic bonds
Glycosyl Transferase (GT): 43
formation of glycosidic bonds
Carbohydrate Esterase (CE): 3
hydrolysis of carbohydrate esters
Polysaccharide Lyase (PL): 0
non-hydrolytic cleavage of glycosidic bonds
Carbohydrate Sulfatases: 2
remove sulfate groups from sulfated glycans
....
B
Plant polysaccharide-degrading GH
Padj < 0.05
Xylan
Endo-1,4-β-xylanase
Glucuronoarabinoxylan endo-1,4-beta-xylanase
Oligosaccharide reducing-end xylanase
α-Glucuronidase
Cellulose
Endo-1,4-β-glucanase
Starch
α-amylase
Pullulanase
Neopullulanase
Mannan
Mannan endo-1,4-β-mannosidase
β-mannosidase
GBC
LF
WF
z score
C
Host glycoprotein-targeting GH
Padj < 0.05
Exo-α-sialidase
Endo-β-N-acetylglucosaminidase
Hyaluronidase
Alpha-N-acetylglucosaminidase
GBC
LF
WF
z score
D
Bacterial intracellular oligosaccharide-metabolizing GH
Padj < 0.05
Maltose-6'-phosphate glucosidase
Malto-oligosyltrehalose trehalohydrolase
Glucan 1,6-α-glucosidase
6-phospho-β-galactosidase
6-phospho-β-glucosidase
GBC
LF
WF
z score

Supplementary Figure 3. PICRUSt2 of 16S rRNA-seq data predicts enrichment of glycoside hydrolases (Related to Figure 2).

Phylogenetic Investigation of Communities by Reconstruction of Unobserved States 2 (PICRUSt2) was performed to predict metagenomes from 16S rRNA-seq data.

(A) Heatmap showing the mean z-scores of predicted enzyme commission (EC) abundances and the number of significantly altered CAZyme ECs across GBC (n=5), LF (n=4), and WF (n=8).

(B-D) Heatmaps of highlighting plant polysaccharide-degrading glycoside hydrolases (GH), host glycoprotein-targeting GH, and bacterial intracellular oligosaccharide-metabolizing GH among significantly altered GH.

Statistical significance was determined by Kruskal–Wallis tests, followed by false discovery rate (FDR) correction ($P_{adj} < 0.05$).

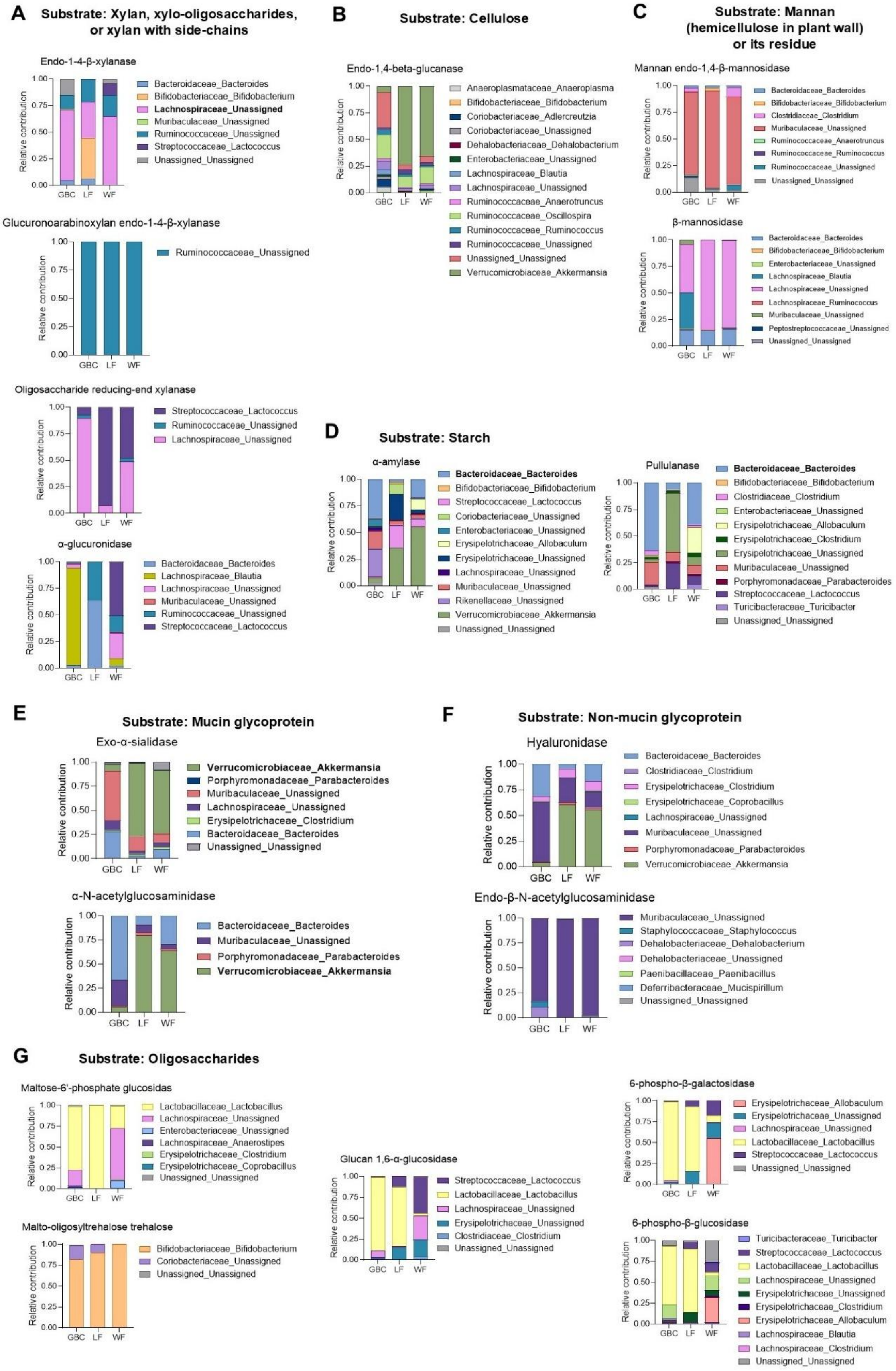
A
Substrate: Xylan, xylo-oligosaccharides, or xylan with side-chains
Endo-1-4-β-xylanase
Bacteroidaceae_Bacteroides
Bifidobacteriaceae_Bifidobacterium
Lachnospiraceae_Unassigned
Muribaculaceae_Unassigned
Ruminococcaceae_Unassigned
Streptococcaceae_Lactococcus
Unassigned_Unassigned
Glucuronoarabinoxylan endo-1-4-β-xylanase
Ruminococcaceae_Unassigned
Oligosaccharide reducing-end xylanase
Streptococcaceae_Lactococcus
Ruminococcaceae_Unassigned
Lachnospiraceae_Unassigned
α-glucuronidase
Bacteroidaceae_Bacteroides
Lachnospiraceae_Blautia
Lachnospiraceae_Unassigned
Muribaculaceae_Unassigned
Ruminococcaceae_Unassigned
Streptococcaceae_Lactococcus
B
Substrate: Cellulose
Endo-1,4-beta-glucanase
Anaeroplasmataceae_Anaeroplasma
Bifidobacteriaceae_Bifidobacterium
Coriobacteriaceae_Adlercreutzia
Coriobacteriaceae_Unassigned
Dehalobacteriaceae_Dehalobacterium
Enterobacteriaceae_Unassigned
Lachnospiraceae_Blautia
Lachnospiraceae_Unassigned
Ruminococcaceae_Anaerotruncus
Ruminococcaceae_Oscillospira
Ruminococcaceae_Ruminococcus
Ruminococcaceae_Unassigned
Unassigned_Unassigned
Verrucomicrobiaceae_Akkermansia
C
Substrate: Mannan (hemicellulose in plant wall) or its residue
Mannan endo-1,4-β-mannosidase
Bacteroidaceae_Bacteroides
Bifidobacteriaceae_Bifidobacterium
Clostridiaceae_Clostridium
Muribaculaceae_Unassigned
Ruminococcaceae_Anaerotruncus
Ruminococcaceae_Ruminococcus
Ruminococcaceae_Unassigned
Unassigned_Unassigned
β-mannosidase
Bacteroidaceae_Bacteroides
Bifidobacteriaceae_Bifidobacterium
Enterobacteriaceae_Unassigned
Lachnospiraceae_Blautia
Lachnospiraceae_Unassigned
Lachnospiraceae_Ruminococcus
Muribaculaceae_Unassigned
Peptostreptococcaceae_Unassigned
Unassigned_Unassigned
D
Substrate: Starch
α-amylase
Bacteroidaceae_Bacteroides
Bifidobacteriaceae_Bifidobacterium
Streptococcaceae_Lactococcus
Coriobacteriaceae_Unassigned
Enterobacteriaceae_Unassigned
Erysipelotrichaceae_Allobaculum
Erysipelotrichaceae_Unassigned
Lachnospiraceae_Unassigned
Muribaculaceae_Unassigned
Rikenellaceae_Unassigned
Verrucomicrobiaceae_Akkermansia
Unassigned_Unassigned
Pullulanase
Bacteroidaceae_Bacteroides
Bifidobacteriaceae_Bifidobacterium
Clostridiaceae_Clostridium
Enterobacteriaceae_Unassigned
Erysipelotrichaceae_Allobaculum
Erysipelotrichaceae_Clostridium
Erysipelotrichaceae_Unassigned
Muribaculaceae_Unassigned
Porphyromonadaceae_Parabacteroides
Streptococcaceae_Lactococcus
Turicibacteraceae_Turicibacter
Unassigned_Unassigned
E
Substrate: Mucin glycoprotein
Exo-α-sialidase
Verrucomicrobiaceae_Akkermansia
Porphyromonadaceae_Parabacteroides
Muribaculaceae_Unassigned
Lachnospiraceae_Unassigned
Erysipelotrichaceae_Clostridium
Bacteroidaceae_Bacteroides
Unassigned_Unassigned
α-N-acetylglucosaminidase
Bacteroidaceae_Bacteroides
Muribaculaceae_Unassigned
Porphyromonadaceae_Parabacteroides
Verrucomicrobiaceae_Akkermansia
F
Substrate: Non-mucin glycoprotein
Hyaluronidase
Bacteroidaceae_Bacteroides
Clostridiaceae_Clostridium
Erysipelotrichaceae_Clostridium
Erysipelotrichaceae_Coprobacillus
Lachnospiraceae_Unassigned
Muribaculaceae_Unassigned
Porphyromonadaceae_Parabacteroides
Verrucomicrobiaceae_Akkermansia
Endo-β-N-acetylglucosaminidase
Muribaculaceae_Unassigned
Staphylococcaceae_Staphylococcus
Dehalobacteriaceae_Dehalobacterium
Dehalobacteriaceae_Unassigned
Paenibacillaceae_Paenibacillus
Deferribacteraceae_Mucispirillum
Unassigned_Unassigned
G
Substrate: Oligosaccharides
Maltose-6'-phosphate glucosidas
Lactobacillaceae_Lactobacillus
Lachnospiraceae_Unassigned
Enterobacteriaceae_Unassigned
Lachnospiraceae_Anaerostipes
Erysipelotrichaceae_Clostridium
Erysipelotrichaceae_Coprobacillus
Unassigned_Unassigned
Malto-oligosyltrehalose trehalose
Bifidobacteriaceae_Bifidobacterium
Coriobacteriaceae_Unassigned
Unassigned_Unassigned
Glucan 1,6-α-glucosidase
Streptococcaceae_Lactococcus
Lactobacillaceae_Lactobacillus
Lachnospiraceae_Unassigned
Erysipelotrichaceae_Unassigned
Clostridiaceae_Clostridium
Unassigned_Unassigned
6-phospho-β-galactosidase
Erysipelotrichaceae_Allobaculum
Erysipelotrichaceae_Unassigned
Lachnospiraceae_Unassigned
Lactobacillaceae_Lactobacillus
Streptococcaceae_Lactococcus
Unassigned_Unassigned
6-phospho-β-glucosidase
Turicibacteraceae_Turicibacter
Streptococcaceae_Lactococcus
Lactobacillaceae_Lactobacillus
Lachnospiraceae_Unassigned
Erysipelotrichaceae_Unassigned
Erysipelotrichaceae_Clostridium
Erysipelotrichaceae_Allobaculum
Lachnospiraceae_Blautia
Lachnospiraceae_Clostridium
Unassigned_Unassigned
Relative contribution
GBC
LF
WF

Supplementary Figure 4. Taxonomic contribution to glycoside hydrolases predicted by PICRUSt2 (Related to Figure 2).

Relative contribution analysis of microbial taxa to glycoside hydrolases predicted by PICRUSt2 among GBC (n=5), LF (n=4), and WF (n=8). The stacked bar plots illustrate contributions to plant polysaccharide-degrading enzymes targeting different substrates (A-D), host glycoprotein-targeting enzymes (E and F), and bacterial intracellular oligosaccharide-metabolizing enzymes (G).

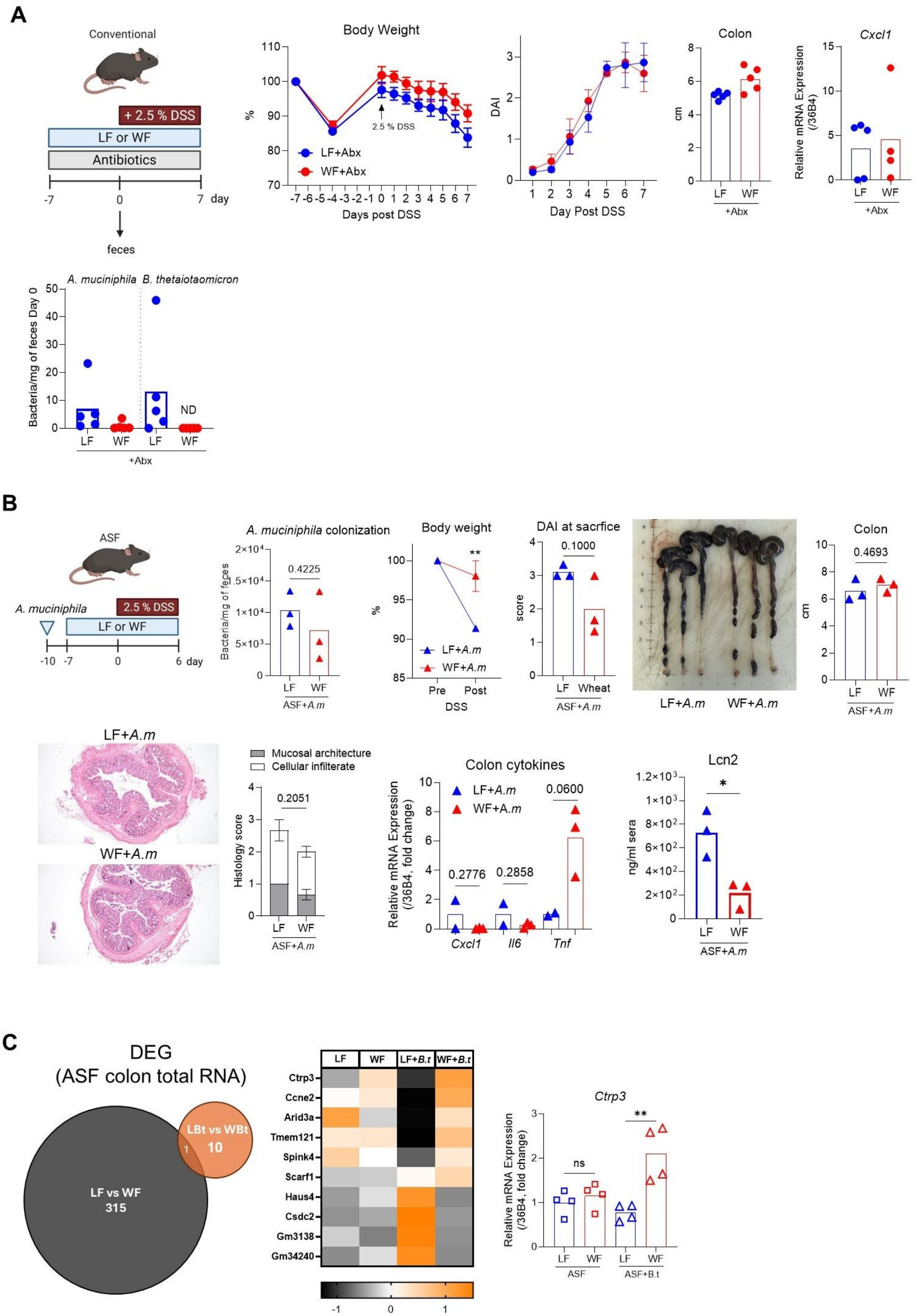
A
Conventional
+ 2.5 % DSS
LF or WF
Antibiotics
-7
0
7 day
feces
Body Weight
%
2.5 % DSS
LF+Abx
WF+Abx
Days post DSS
DAI
Day Post DSS
Colon
cm
LF
WF
+Abx
Cxcl1
Relative mRNA Expression (/36B4)
A. muciniphila
B. thetaiotaomicron
Bacteria/mg of feces Day 0
ND
B
ASF
A. muciniphila
2.5 % DSS
LF or WF
-10
-7
0
6 day
A. muciniphila colonization
Bacteria/mg of feces
0.4225
ASF+A.m
Body weight
**
LF+A.m
WF+A.m
Pre
Post
DSS
DAI at sacrfice
0.1000
score
Wheat
Colon
0.4693
Mucosal architecture
Cellular infiltrate
0.2051
Histology score
Colon cytokines
Relative mRNA Expression (/36B4, fold change)
0.2776
0.2858
0.0600
Cxcl1
Il6
Tnf
Lcn2
*
ng/ml sera
C
DEG
(ASF colon total RNA)
LBt vs WBt
10
1
LF vs WF
315
LF
WF
LF+B.t
WF+B.t
Ctrp3
Ccne2
Arid3a
Tmem121
Spink4
Scarf1
Haus4
Csdc2
Gm3138
Gm34240
-1
0
1
Ctrp3
ns
**
ASF
ASF+B.t

Supplementary Figure 5. WF's protection required complex microbiota and *Akkermansia* moderately restored the WF-mediated colitis protection (Related to Figure 2).

(A) Conventional mice (n=5/group) were fed either LF or WF for one week, followed by DSS water for 7 days. Conventional mice were given an antibiotics cocktail (ampicillin, neomycin, and metronidazole; 1 g/L each) in drinking water throughout the experiment. Body weight and DAI were monitored, and colon length and colon *Cxcl1* were measured. Total bacterial load and absolute abundances of *A. muciniphila* and *B. theta* prior to DSS were quantified by qPCR.

(B) ASF mice (n=3/group) were gavaged with *A. muciniphila* under normal chow, then switched to either LF or WF in 3 days for one week, followed by DSS water for 6 days. Body weights before and after DSS treatment and DAI on the day of sacrifice. Serum Lcn2 levels measured by ELISA. Representative colon images and colon length measurements. Colon inflammatory cytokine transcript levels measured by RT-qPCR. Representative colon histology images and scoring.

(C) Differentially expressed genes analyzed from total mRNA sequencing on colons of ASF mice fed LF or WF with or without *B. theta.* Heaptmap on 10 DEGs between LF+B.t and WF+B.t. *Ctrp3* expression by qPCR.

All data are presented as mean values ±SEM. Statistical significance was assessed using unpaired two tailed t-test (B), two-way ANOVA followed by Šídák's multiple comparisons test (B), or one-way ANOVA followed by Tukey's multiple comparisons test (C). ns P > 0.05, *P < 0.05, **P < 0.01, **P < 0.01.

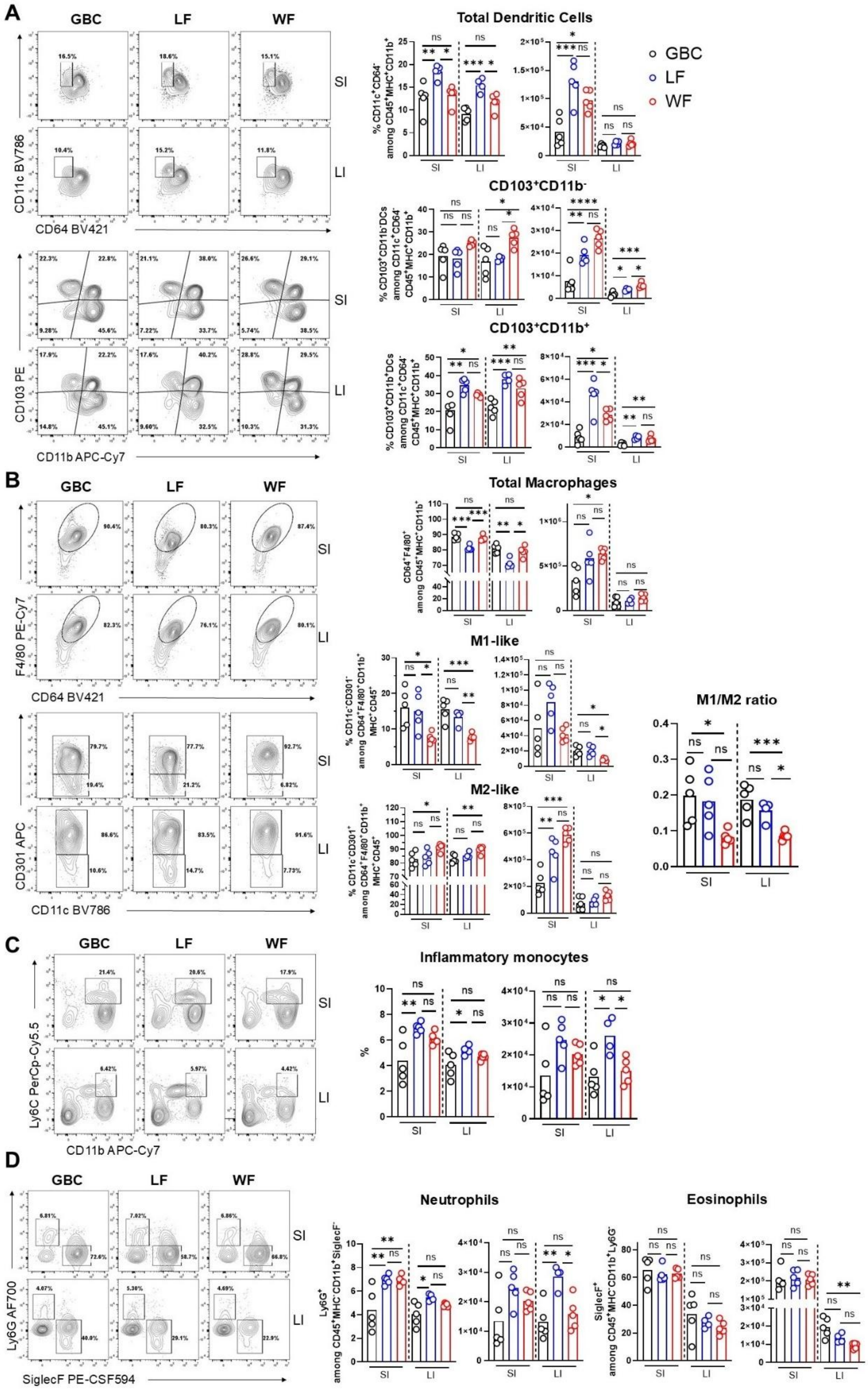
A
GBC
LF
WF
SI
LI
CD11c BV786
CD64 BV421
CD103 PE
CD11b APC-Cy7
Total Dendritic Cells
CD103+CD11b-
CD103+CD11b+
B
F4/80 PE-Cy7
CD301 APC
CD11c BV786
Total Macrophages
M1-like
M2-like
M1/M2 ratio
C
Ly6C PerCp-Cy5.5
Inflammatory monocytes
D
Ly6G AF700
SiglecF PE-CSF594
Neutrophils
Eosinophils

Supplementary Figure 6. WF altered the basal inflammatory tone in the gut (Related to Figure 3A).

WT conventional mice (n=5) were fed either GBC, LF, or WF for one week. The lamina propria of the small and large intestines was analyzed by flow cytometry to assess innate immune cells, including (A) dendritic cells, (B) macrophages, (C) inflammatory monocytes, and (D) neutrophils and eosinophils. All data are presented as mean values $\pm$SEM. Statistical significance was assessed using one-way ANOVA followed by Tukey's multiple comparison test. ns $P > 0.05$, *$P < 0.05$, **$P < 0.01$, ***$P < 0.001$.

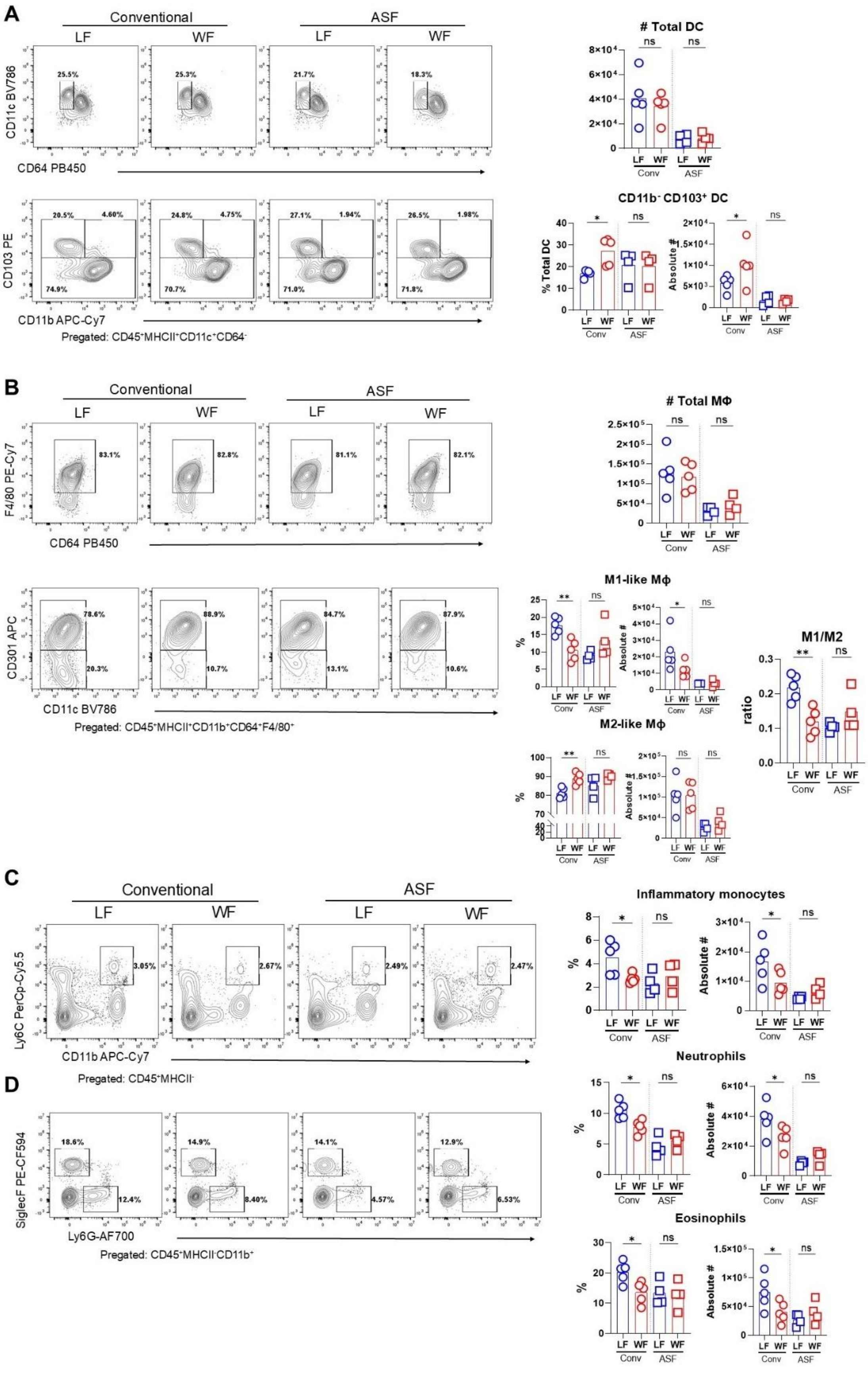

A
Conventional
ASF
LF
WF
CD11c BV786
CD64 PB450
CD103 PE
CD11b APC-Cy7
Pregated: CD45+MHCII+CD11c+CD64-
Total DC
CD11b- CD103+ DC
% Total DC
Absolute #
Conv
B
F4/80 PE-Cy7
Total MΦ
M1-like MΦ
M2-like MΦ
M1/M2
ratio
CD301 APC
Pregated: CD45+MHCII+CD11b+CD64+F4/80+
C
Inflammatory monocytes
Ly6C PerCp-Cy5.5
Pregated: CD45+MHCII-
D
Neutrophils
Eosinophils
SiglecF PE-CF594
Ly6G-AF700
Pregated: CD45+MHCII-CD11b+

Supplementary Figure 7. WF-derived changes in innate immune cells are microbiota-dependent (Related to Figure 3A).

B6 conventional and Altered Schaedler Flora (ASF) mice (n=4-5/group) were fed either LF or Wheat for one week. The lamina propria of the colon was analyzed by flow cytometry to assess innate immune cells, including (A) dendritic cells, (B) M1 and M2 macrophages, (C) inflammatory monocytes, and (D) neutrophils and eosinophils. All data are presented as mean values $\pm$SEM. Statistical significance was assessed using one-way ANOVA followed by Šídák's multiple comparisons test. ns $P > 0.05$, $^{*}P < 0.05$, $^{**}P < 0.01$.

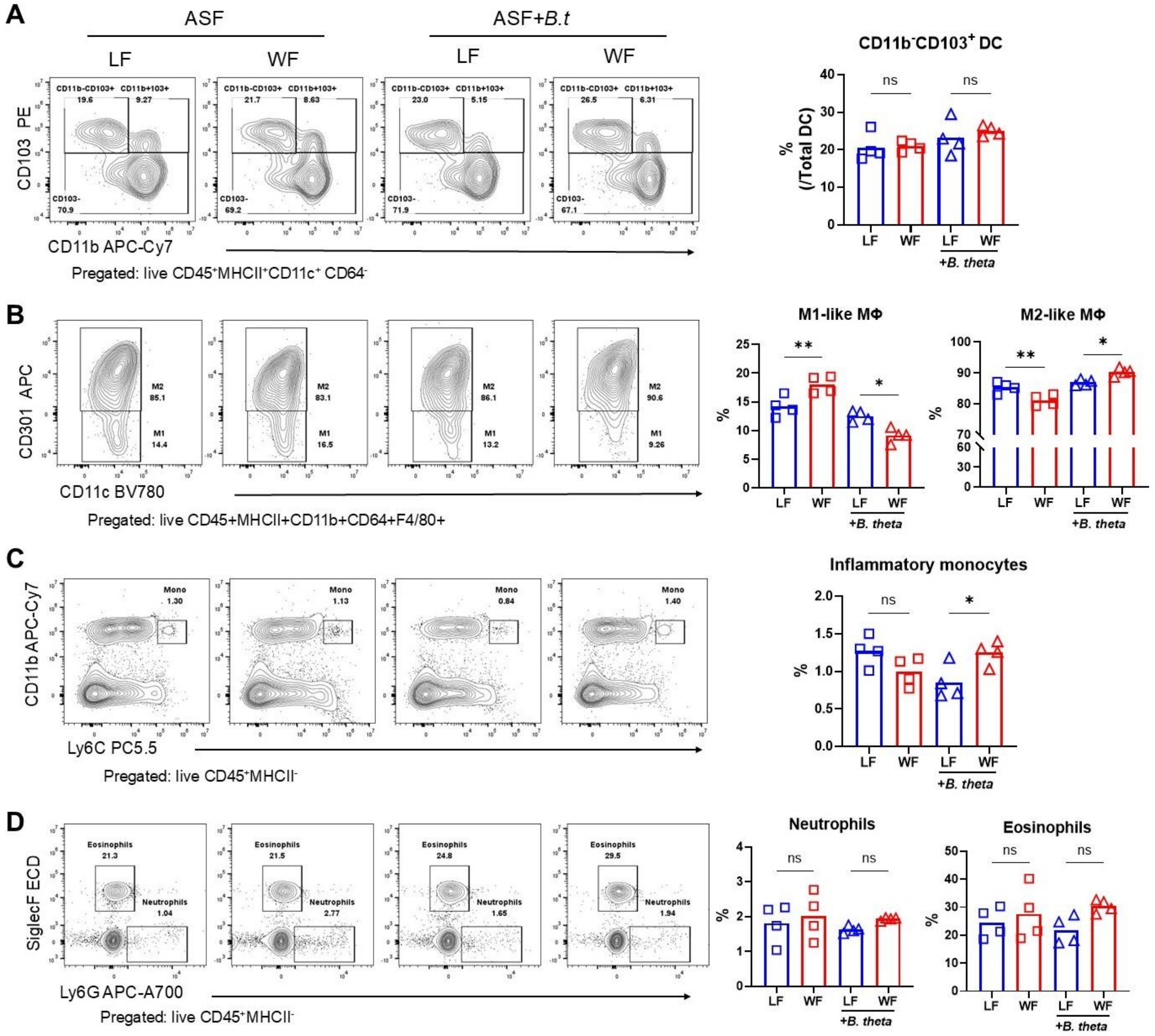

Supplementary Figure 8. *B. theta* didn't restore the WF-induced changes in innate immune cells in the colon of ASF (Related to Figure 3B).

ASF mice (n=4/group) were fed either LF or WF for one week. Half of the mice were gavaged with *B.theta* three days prior to the dietary intervention. (A) CD103$^+$CD11b$^-$ DC, (B) Macrophages, (C) inflammatory monocytes, (D) neutrophils, and eosinophils from the colon lamina propria was analyzed by flow cytometry. All data are presented as mean values ±SEM. Statistical significance was assessed using one-way ANOVA followed by Šídák's multiple comparisons test. ns P > 0.05, *P < 0.05, **P < 0.01.

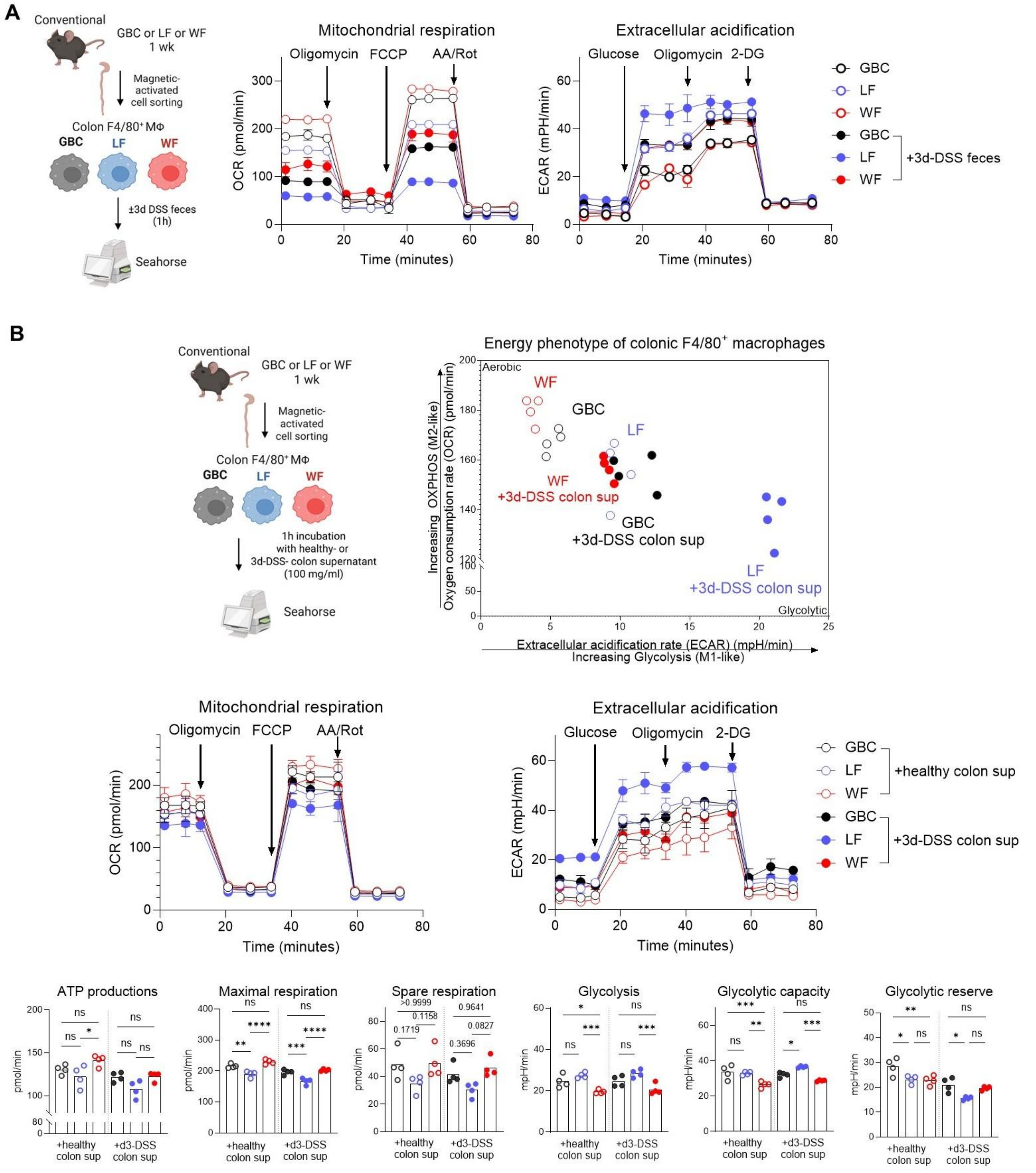

Supplementary Figure 9. Seahorse analysis on diet-fed colon macrophages in response to inflammatory stimuli (Related to Figure 3C).

Conventional B6 mice (n=4/group) were fed GBC, LF, or WF for 1 week. F4/80+ colon macrophages were isolated by beads and their cellular metabolism was analyzed by Seahorse after being incubated (37 °C, 1 hour) with feces (A) or colon supernatant (100 mg/ml in PBS) (B) from day 3 DSS-treated mice.

All data are presented as mean values $\pm$SEM. Statistical significance was assessed using one-way ANOVA followed by Šídák's multiple comparisons test. ns $P > 0.05$, *$P < 0.05$, **$P < 0.01$, *** $P < 0.001$.

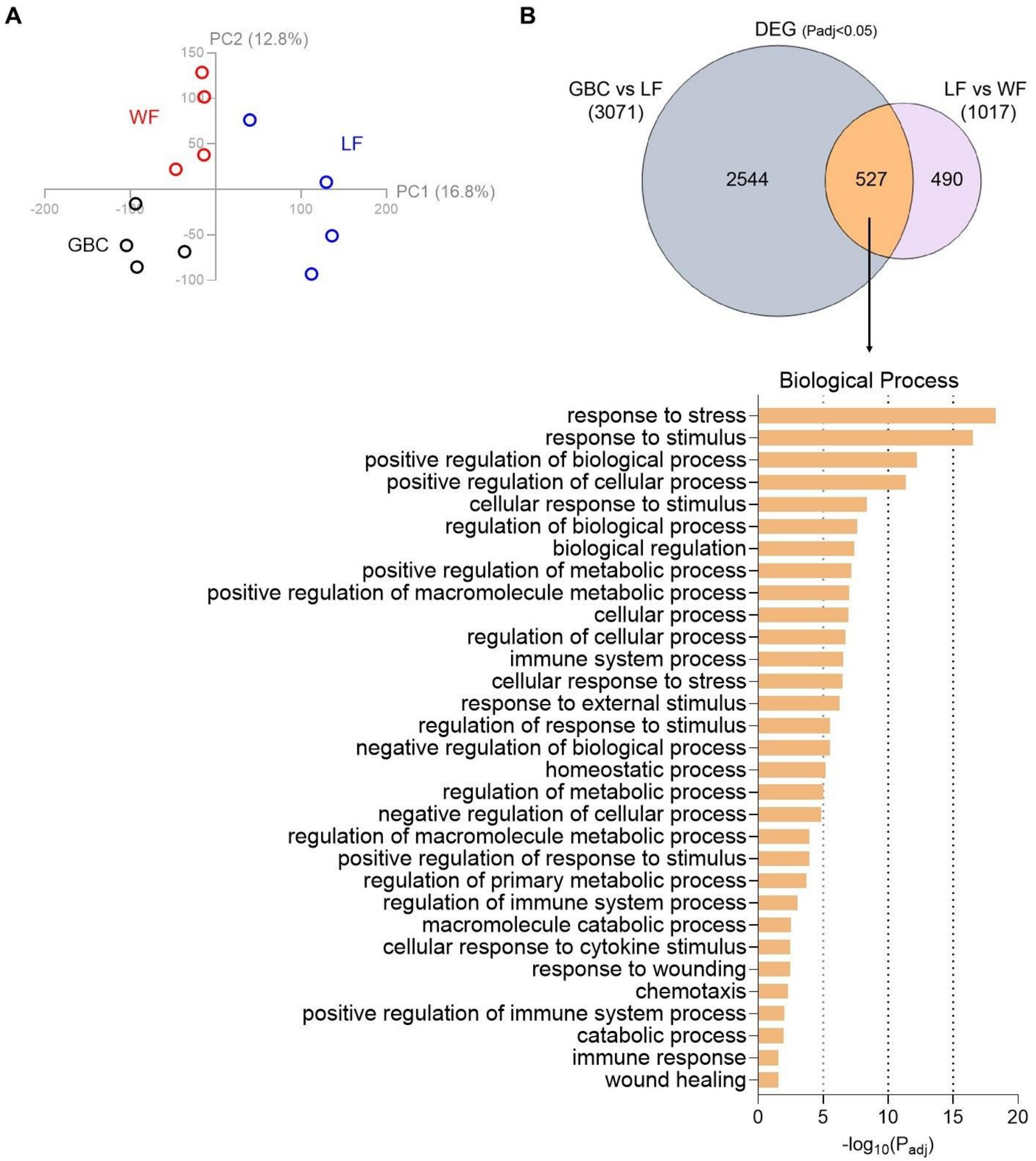

Supplementary Figure 10. Colon macrophages mRNA-seq (Related to Figure 3D).

(A) mRNA-seq was performed on colon F4/80+ macrophages from mice fed GBC, LF, or WF. PCA plot was plotted from normalized gene counts post DESeq2 analysis.

(B) The number of DEG without fold-change criteria and the GO enrichment analysis result on overlapping 527 genes between GBC-LF DEG and WF-LF DEG.

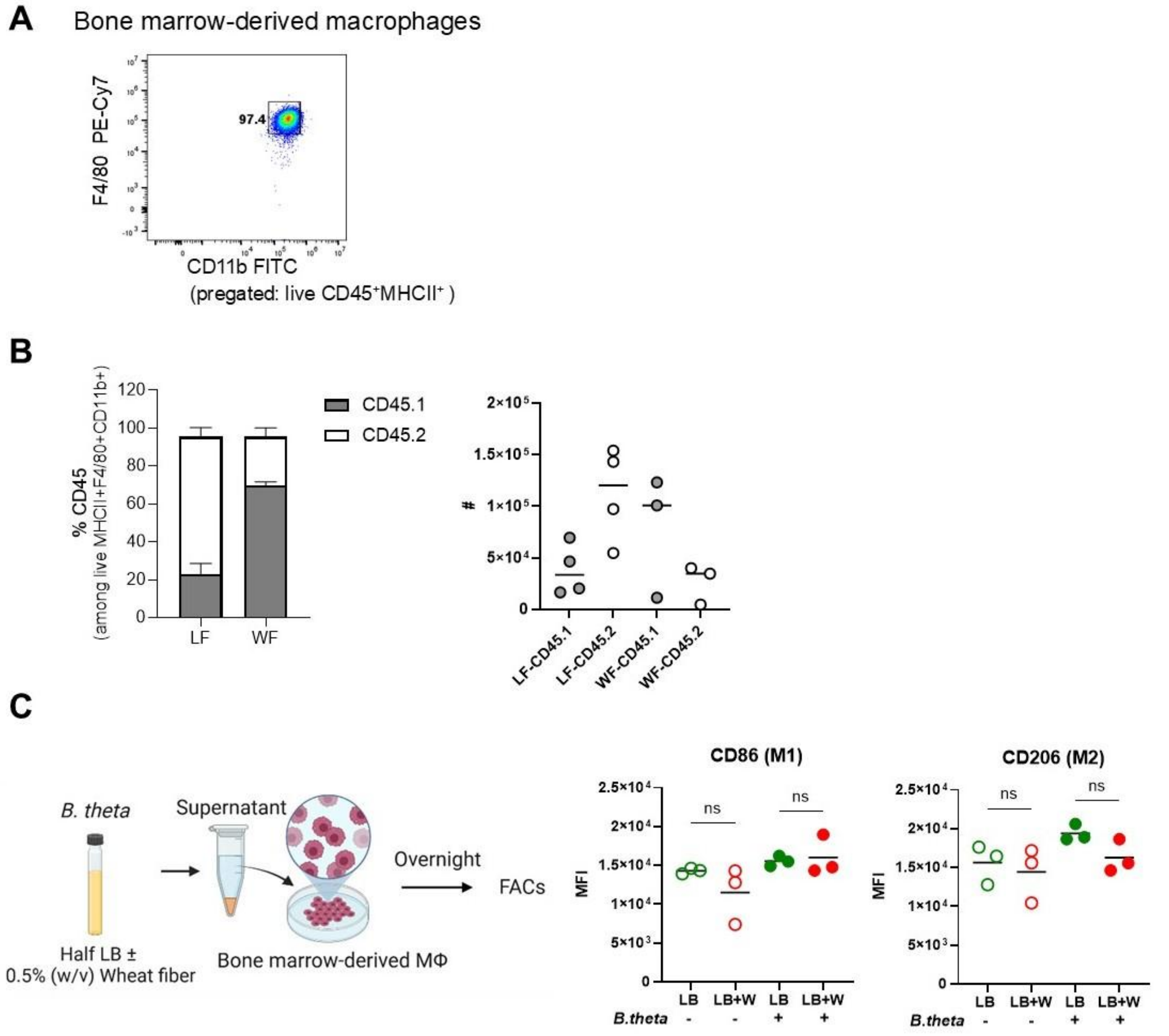

Supplementary Figure 11. *B. theta* culture supernatant failed to induce WF-derived M1/M2 phenotype in BMDM (Related to Figure 4).

(A) FACs plot bone marrow-derived macrophages after 7-day differentiation from bone marrow.

(B) CD45.2 mice (n=4/group) were fed with LF for 1 week and given DSS water for 5 days. One day before DSS treatment, LF or WF FS-treated CD45.1 BMDM (5x10^5 cells/mouse) were intraperitoneally injected. Upon sacrifice, relative CD45 ratio among macrophages and their absolute number were analyzed by flow cytometry.

(C) *B. theta* were cultured in LB broth with or without 0.5% (w/w) WF. Media control and *B. theta* culture were collected, and supernatants were used to treat BMDM overnight for supernatant. M1/M2 polarization was measured by flow cytometry.

All data are presented as mean values ±SEM. Statistical significance was assessed using one-way ANOVA followed by Sidak's multiple comparisons test. ns P > 0.05

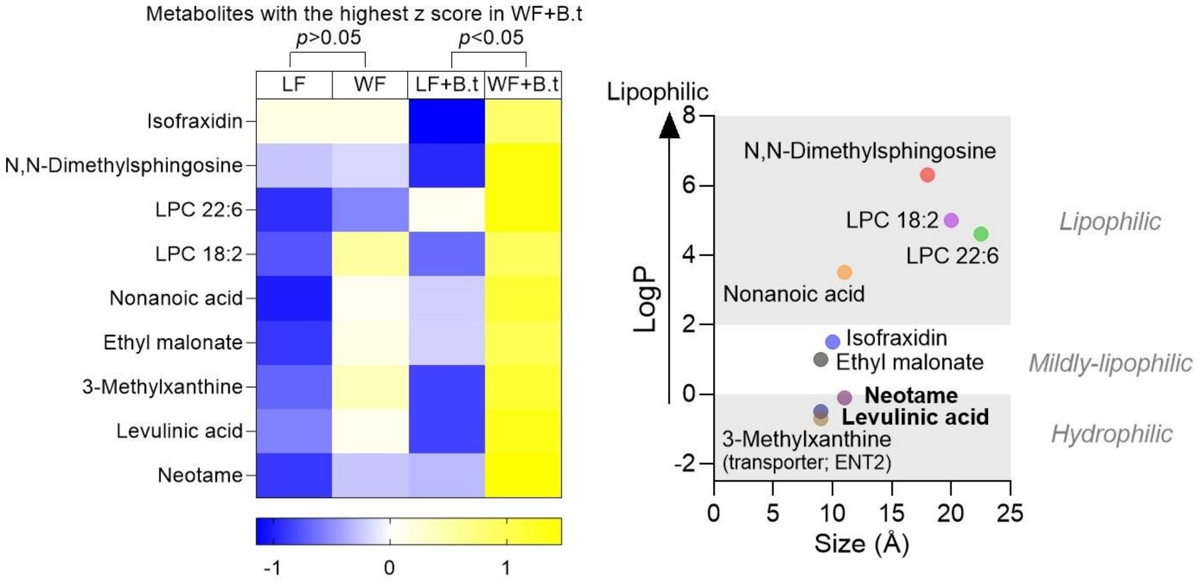

Supplementary Figure 12. Two hydrophilic metabolites, although associated with WF and *B. theta*, were excluded from consideration as potential metabolites capable of reaching the colon lamina propria (Related to Figure 5).

Fecal supernatants of ASF mice (n=4/group) fed either LF or WF and colonized with or without *B.theta* were analyzed by untargeted metabolomics. Heatmap shows top metabolites with the highest z-scores in WF+B.t, excluding those without significant differences between WF+B.t and LF+B.t. LogP value (Octanol–water partition coefficient; lipophilicity) for each molecule can be found in PubChem.

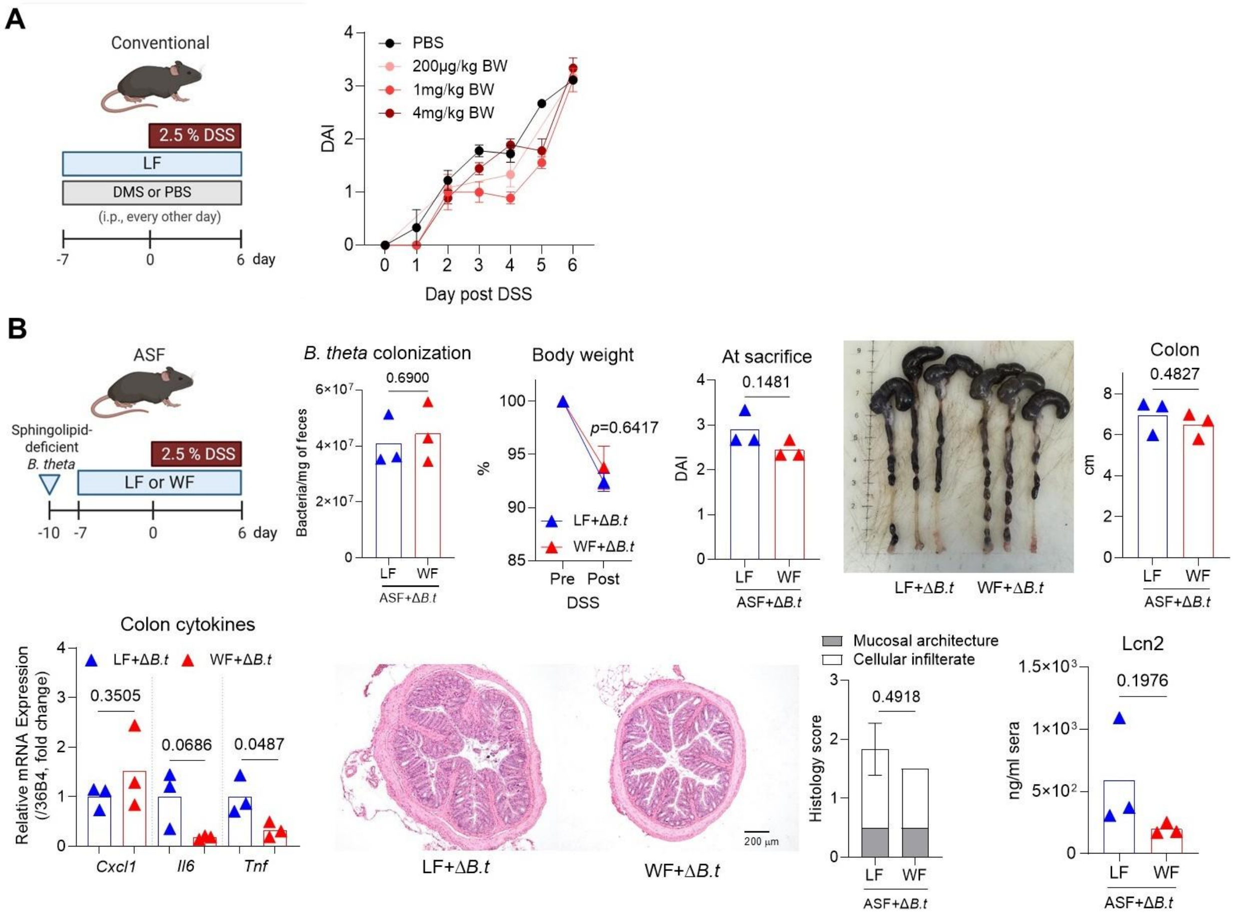

Supplementary Figure 13. DMS administration didn't protect mice against DSS colitis but sphingolipid mutant *B. theta* failed to protect mice in ASF (Related to Figure 5).

(A) Conventional mice (n=4/group) were intraperitoneally injected with DMS (0.2, 1, or 4 mg/kg bw) or PBS while on LF, and then challenged with 2.5% DSS water for 6 days. DAI post DSS was monitored.

(B) ASF mice (n=3/group) were gavaged with serine palmitoyl transferase (SPT) mutant *B. theta* under normal chow, then switched to either LF or WF after 3 days for a week, followed by DSS water for 6 days. *B. theta* colonization was confirmed by qPCR. Body weights before and after DSS. DAI at sacrifice. Representative colon images and colon length measurements. Colon inflammatory cytokine transcript levels measured by RT-qPCR. Representative colon histology images and scoring. Serum Lcn2 levels measured by ELISA.

All data are presented as mean values ±SEM. Statistical significance was assessed using unpaired two-tailed t-test or two-way ANOVA followed by Šídák's multiple comparisons test (Body weight).

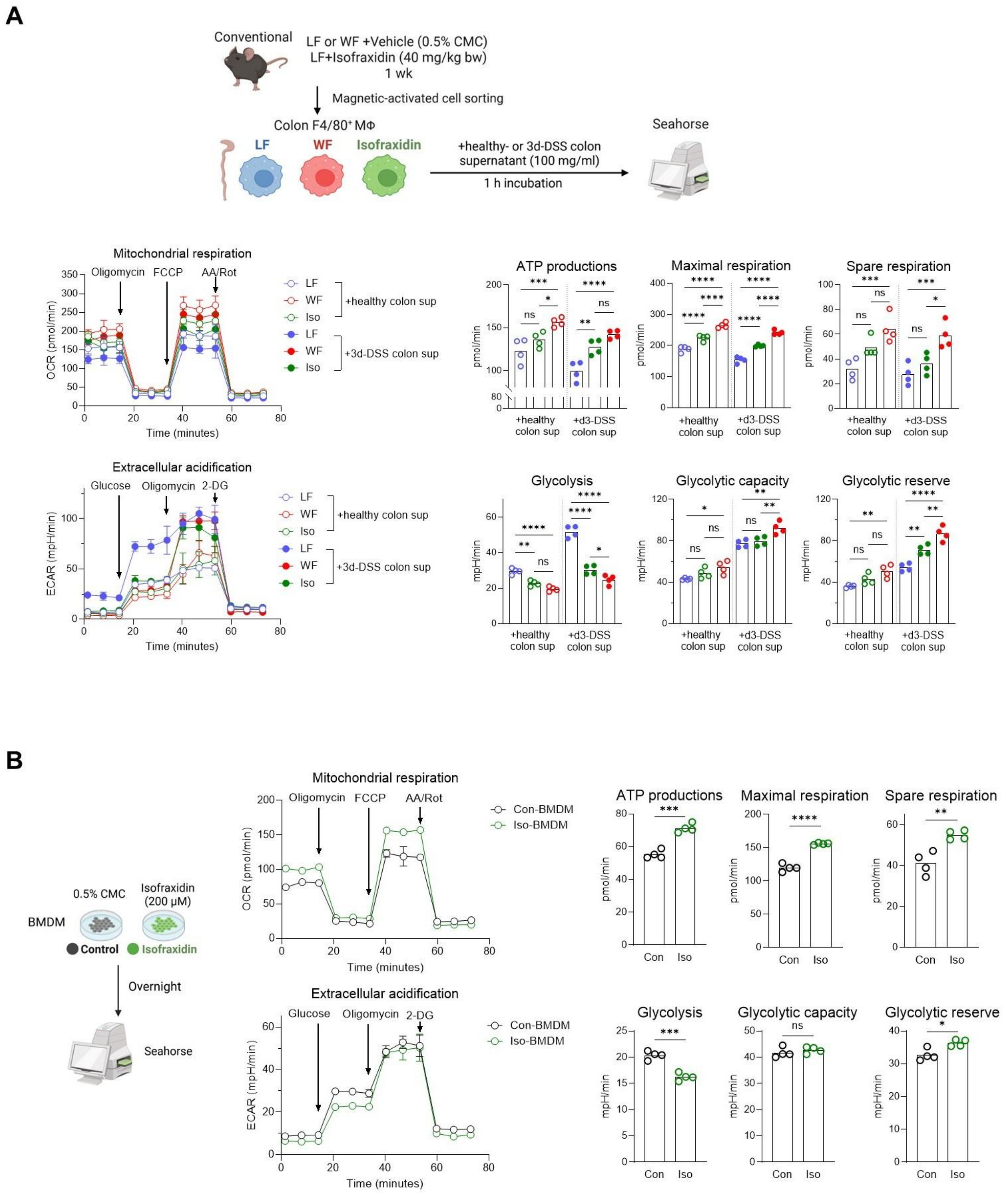

Supplementary Figure 14. Isofraxidin altered the cellular metabolism of macrophages *in vivo and in vitro* (Related to Figure 5).

(A) Conventional mice (n=4/group) were gavaged with isofraxidin (40 mg/kg bw) on LF or 0.5% CMC while on LF or WF. Then colon F4/80$^+$ macrophages were isolated using beads and their

cellular metabolism was analyzed by Seahorse after being incubated (37 °C, 1 hour) with colon supernatant (100 mg/ml in PBS) from healthy or day 3 DSS-treated mice.

(B) BMDM were treated overnight either with isofraxidin (200 μM) or 0.5% CMC and their cellular metabolism was analyzed by Seahorse.

All data are presented as mean values ±SEM. Statistical significance was assessed using one-way ANOVA followed by Sidak's multiple comparions test (A), or unpaired two-tailed t-test (B). ns $P > 0.05$, *$P < 0.05$, ** $P < 0.01$, *** $P < 0.001$, **** $P < 0.0001$.
